# Inulin Diet Alleviates Abdominal Aortic Aneurysm by Increasing Akkermansia and Improving Intestinal Barrier

**DOI:** 10.3390/biomedicines13040920

**Published:** 2025-04-09

**Authors:** Shuang Guo, Fen Yang, Jiyu Zhang, Yuhan Liao, Ni Xia, Tingting Tang, Chaolong Wang, Qing K. Wang, Chen Chen, Desheng Hu, Zhilei Shan, Xiang Cheng

**Affiliations:** 1 Department of Cardiology, Union Hospital, Tongji Medical College, Huazhong University of Science and Technology, Wuhan 430074, China; gsa03649@btch.edu.cn (S.G.); yangfen913@163.com (F.Y.); d202081671@hust.edu.cn (J.Z.);; 2 Hubei Key Laboratory of Biological Targeted Therapy, Union Hospital, Tongji Medical College, Huazhong University of Science and Technology, Wuhan 430074, China; 3 Hubei Engineering Research Center for Immunological Diagnosis and Therapy of Cardiovascular Diseases, Union Hospital, Tongji Medical College, Huazhong University of Science and Technology, Wuhan 430074, China; 4 Department of Vascular Surgery, Beijing Tsinghua Changgung Hospital, School of Clinical Medicine, Tsinghua University, Beijing 100084, China; 5 Department of Epidemiology and Biostatistics, School of Public Health, Tongji Medical College, Huazhong University of Science and Technology, Wuhan 430074, China; 6 Center for Human Genome Research, Key Laboratory of Molecular Biophysics of the Ministry of Education, College of Life Science and Technology, Huazhong University of Science and Technology, Wuhan 430074, China; 7 Division of Cardiology, Department of Internal Medicine, Tongji Hospital, Tongji Medical College, Huazhong University of Science and Technology, Wuhan 430074, China; 8 Department of Integrated Traditional Chinese and Western Medicine, Union Hospital, Tongji Medical College, Huazhong University of Science and Technology, Wuhan 430074, China; 9 Department of Nutrition and Food Hygiene, Hubei Key Laboratory of Food Nutrition and Safety, School of Public Health, Tongji Medical College, Huazhong University of Science and Technology, Wuhan 430074, China

**Keywords:** aneurysm, high-fiber diet, microbiota, intestinal barrier, Ly6Chi monocyte

## Abstract

**Background/Objectives**: Previous studies have shown varying efficacy of high-fiber diets containing different ingredients in abdominal aortic aneurysms (AAAs). This study aimed to identify which high-fiber diet protects against AAA in mice and elucidate the underlying mechanisms. **Methods**: This study compared inulin, cellulose, and chow diets in terms of their impact on aneurysm enlargement, elastin degradation, matrix metalloproteinase 2 and 9 expressions, CD3+ T cell and CD68+ macrophage infiltration, and macrophage differentiation. It also examined gut microbiota composition, focusing on Akkermansia, and evaluated intestinal barrier function and systemic inflammatory response. **Results**: The inulin diet, but not the cellulose diet, compared with the chow diet, reduced aneurysm enlargement, elastin degradation, matrix metalloproteinase 2 and 9 expressions, CD3+ T cell and CD68+ macrophage infiltration, and skewed macrophage towards M2 differentiation. The inulin diet enriched Akkermansia in both the small and large intestine. The inulin diet also enhanced the intestinal barrier by augmenting goblet cells, upregulating the gene related to the epithelial barrier and antibacterial peptides in the small intestine, and reducing circulating lipopolysaccharide and interleukin-1β levels. The inulin diet lowered the proportion of Ly6Chi monocytes and C-C chemokine receptor 2 expression on these cells in the bone marrow, reducing aneurysm infiltration. Administering Akkermansia to AAA mice decreased intestinal permeability and mitigated AAA. **Conclusions**: A diet rich in fermentable fiber inulin, as opposed to cellulose, alleviates AAA in mice. This beneficial effect is attributed to the enhanced presence of Akkermansia bacteria and improvement of the intestinal barrier.

## 1. Introduction

Abdominal aortic aneurysm (AAA) is a chronic vascular inflammatory disease characterized by the asymptomatic and fatal permanent dilation of the abdominal aorta, with a mortality exceeding 80% in cases of rupture [1]. The pathological features of AAA primarily include vascular smooth muscle cell apoptosis, elastin degradation, and infiltration of inflammatory cells. These features are often accompanied by arterial wall calcification and the formation of intraluminal thrombi [2]. Pro-inflammatory monocytes/macrophages and T cells play essential roles in the pathophysiology of AAA [2,3]. Monocytes expressing C-C motif chemokine receptor (CCR) 2 are recruited to the aneurysm and differentiate into macrophages. These macrophages exacerbate the aortic dilation by secreting proteases, specifically matrix metalloproteinases (MMP) 2 and 9, along with cytokines and chemokines, creating an imbalanced pro-inflammatory microenvironment that worsens AAA [4].

The current primary treatments for AAA include open surgery and endovascular aneurysm repair, the latter being reserved for patients with significant dilation or rapid progression. Various medications, including statins, metformin, antihypertensive drugs, and anti-inflammatory agents, have been investigated in animal models [5,6]. However, no medication has demonstrated consistent and significant efficacy in randomized clinical trials [7]. The absence of effective non-surgical treatments underscores the importance of developing medication to enhance long-term prognosis and guide the clinical management of the disease.

Previous epidemiological studies have demonstrated an inverse relationship between high-fiber diets and the occurrence of cardiovascular diseases, such as AAA [8,9]. Cohort studies have shown that individuals who consume high-fiber diets exhibit a decreased risk of AAA. Furthermore, the hazard ratios pertaining to various types of fiber intake have shown variability [10].

In accordance with the latest definitions provided by the World Health Organization, dietary fiber includes all carbohydrates or their analogs that resist digestion by enzymes [11]. Natural dietary fiber can be categorized into fermentable, partially fermentable, and nonfermentable fiber. For instance, inulin exemplifies highly fermentable fiber, serving as a substrate for intestinal microbial fermentation, whereas cellulose exemplifies fiber with low fermentability. Unlike mammals, certain species of gut microbiota possess enzymes capable of fermenting dietary fibers into small molecules like short-chain fatty acids (SCFAs) [12], which play a role in modulating microbiota composition and intestinal homeostasis. Moreover, SCFAs also exhibit the potential to mitigate the progression of AAA [13].

We hypothesized that an inulin diet could offer protection against AAA by modulating the intestinal microbiota. Our findings demonstrate that the inulin diet ameliorates AAA by enhancing the abundance of Akkermansia and improving the intestinal barrier. This study establishes a theoretical framework and empirical evidence for the non-surgical management of AAA.

## 2. Materials and Methods

### 2.1. Study Design

SPF C57BL/6 mice were subjected to a 4-week administration of either a chow diet, 15% cellulose diet, or 15% inulin diet before AAA induction through elastase injection. The mice were euthanized for assessment two weeks after AAA induction. To estimate the role of *A. muciniphila* in AAA, the SPF C57BL/6J mice were administered A. muciniphila by gavage every 5 days per week for 2 weeks prior to the induction of AAA using elastase. The administration of *A. muciniphila* continued for two weeks after the induction of AAA. The mice were then euthanized for evaluation.

### 2.2. Animals

Specific pathogen-free (SPF) male C57BL/6J mice, aged 6 weeks and weighing 18–22 g, were purchased from Beijing Vital River Laboratory Animal Technology Co., Ltd. (Beijing, China). The mice were housed in ventilated cages within the SPF facility at Huazhong University of Science and Technology in Wuhan, China. They were randomly allocated to various groups and provided with ad libitum access to food and water according to the specific experimental requirements, while being maintained under a strict 12 h light/dark cycle. Ethical approval for the animal-related procedures was granted by the Animal Care and Utilization Committee of Huazhong University of Science and Technology (no. [2017]-S100), and all procedures were conducted in compliance with the guidelines outlined by National Institutes of Health. The animal caretakers and investigators conducting or assessing the experiments were blinded to the allocation sequence. Sample size was determined with G*power software 3.1 (Heinrich-Heine-Universität Düsseldorf), with an estimated effect size derived from the relevant literature. The minimal sample size (n) was chosen based on ethical considerations.

### 2.3. Diet Administration

Two types of high-fiber diets were prepared by adding either 15% highly fermented fiber inulin or 15% poorly fermented fiber cellulose to the diet (ReadyDietech, Shenzhen, China) [14]. The control diet consisted of a chow diet. Male C57BL/6J mice, aged 6 weeks, were randomly assigned to different groups and given ad libitum access to either the chow diet, 20% inulin high-fiber diet, or 20% cellulose high-fiber diet for a duration of 6 weeks. The diets were changed every three days throughout the course of the experiment.

### 2.4. Elastase-Induced AAA Mouse Model

Male mice were employed for the induction of AAA. The procedure involved the following steps: The mice were initially anesthetized with 4% (*v*/*v*) isoflurane in an induction chamber (RWD; Shenzhen, China). Once the mice showed difficulty standing, 2% (*v*/*v*) isoflurane was delivered via an anesthesia mask. The adequacy of anesthesia was determined by the lack of response to a moderate toe press by an index finger. The mice were securely positioned in a supine posture on a heating pad to maintain warmth throughout the surgery. The abdominal hair was shaved, and an incision was made along the abdominal midline, approximately 1.5 cm in length, through the skin and muscle layers. The contents in the peritoneal cavity were carefully removed to expose the post-peritoneum.

An elastase-induced AAA model was established following previously reported procedures [13]. In brief, the infrarenal region of the abdominal aorta was isolated under a stereomicroscope. Subsequently, a small piece of gauze soaked with 10 μL of porcine pancreatic elastase (E1250, Sigma-Aldrich, St. Louis, MO, USA) was applied around the aorta for 10 min. Similarly, mice in the sham operation group underwent treatment with heat-inactivated elastase for the same duration. After rinsing the peritoneal cavity twice, the muscle and skin layers were closed using interrupted sutures. Buprenorphine was administered subcutaneously every 12 h for the first 48 h post-procedure to alleviate pain. The mice were euthanized 14 days after AAA induction with elastase.

### 2.5. Measurement of Aorta Enlargement

Following sacrifice, blood was flushed out by injecting heparin saline into the left ventricle of each mouse. The abdominal aorta was then carefully separated from the peri-aorta connective tissue and ex vivo images were captured using a Nikon D7200 camera (Tokyo, Japan). To assess aneurysmal dilation, investigators blinded to the treatment groups performed measurements on the maximum external diameter of the infrarenal aorta. The diameter was measured three times for each mouse using ImageJ 1.53t software (NIH, Bethesda, MD, USA), and the mean value was calculated.

### 2.6. EVG Staining and Immunohistochemistry (IHC) Staining

Aneurysms were fixed in 4% paraformaldehyde for 24 h before being embedded in paraffin. To ensure a thorough examination of the pathology, 4 to 8 sections with a 200 μm interval were obtained from each aneurysm, and 4 sections were mounted on a single slide. Each section had a thickness of 4 μm. Paraffin sections were subjected to heat, deparaffinized in xylene, and rehydrated through a series of graded ethanol baths.

EVG staining (115974, Sigma-Aldrich, MO, USA), which stains elastin in a dark color, allowed us to visualize the degradation of elastin. IHC staining was conducted using the following primary antibodies: anti-CD3 (ab5690, 1/100; Abcam, Cambridge, UK), anti-CD68 (ab283654, 1/100; Abcam, Cambridge UK), anti-MMP2 (ab37150, 1/100; Abcam, Cambridge, UK), anti-MMP9 (ab38898, 1/100; Abcam, Cambridge, UK), and IgG control (ab37415; Abcam, Cambridge, UK). RGB images were captured as TIFF files at 10× or 20× magnification using an OLYMPUS BX51 microscope (Olympus; Tokyo, Japan). Image analysis was performed using ImageJ 1.53t (NIH, Bethesda, MD, USA).

Quantitative analysis was conducted by two trained and independent observers who were blinded to the experimental design. The ratio of EVG-positive staining was expressed as the percentage of positively stained area relative to the total sectional aortic area. The quantification of CD68+ macrophages or CD3+ T cells involved counting positively stained cells in each cross-section of the aneurysm. For MMP2 or MMP9 staining, the calculation was based on the ratio of the positive staining area to the total cross-sectional area. The mean value was determined from 4 to 8 sections of each mouse.

### 2.7. Goblet Cell Quantification

Goblet cell quantification was performed following established methods [15]. Tissue sections from the distal small intestine and proximal colon were prepared and stained with periodic acid Schiff–Alcian Blue (PAS-AB) (Solarbio, Beijing, China). Manual counting of goblet cells (GCs) and measurement of the total length of villi were conducted using ImageJ 1.53t (NIH, Bethesda, MD, USA). The goblet cell count was expressed as the number of GCs per μm of villi or mucosal folds. A minimum of 10-15 villi or 3-4 mucosal folds were counted for each replicate.

### 2.8. Sample Collection and Single Cell Isolation

Peripheral blood collection was performed using a pyrogen-free EDTA anticoagulant tube, followed by centrifugation. The upper-layer plasma was stored for the detection of various markers, while the precipitate was resuspended in 2 mL of PBS. Subsequently, the resuspended material was loaded onto 2 mL of lymphocyte separation medium and centrifuged at 800× *g* for 20 min at room temperature with acceleration and no braking.

The spleen was ground, and the bone marrow was rinsed in PBS with 10% fetal bovine serum. Subsequently, red blood cells were lysed using a red blood cell lysis solution (eBioscience, San Diego, CA, USA). The obtained leukocytes were utilized for flow cytometry or cell culture experiments.

The aorta was excised under a stereomicroscope and minced into small pieces in a digestion mixture containing 237 U/mL collagenase I (C0130, Sigma-Aldrich, St. Louis, MO, USA), 190 U/mL collagenase XI (C7657, Sigma-Aldrich, St. Louis, MO, USA), 120 U/mL DNase I (D4527, Sigma-Aldrich, St. Louis, MO, USA), and 120 U/mL hyaluronidase (H3506, Sigma-Aldrich, St. Louis, MO, USA). The tissue was then incubated three times at 37 °C with 120 r.p.m. for 20 min each. The resulting digestion solution was collected and filtered through a 40 µm microfilter for subsequent flow cytometry staining and analysis [13].

### 2.9. Flow Cytometry Staining and Analysis

Cell samples were stained with fluorochrome-conjugated antibodies against surface markers to identify populations of interest. After staining for surface markers in PBS at 4 °C for 30 min, the cells were then fixed. The following antibodies were used: PEcy7-labeled anti-Ly6c (128018, BioLegend, San Diego, CA, USA), PerCP/cy5.5-labeled anti-CD11b (101228, BioLegend, San Diego, CA, USA), FITC-labeled anti-CD45 (103108, BioLegend, San Diego, CA, USA), APC-cy7-labeled anti-Ly6G (127624, BioLegend, San Diego, CA, USA), APC-labeled anti-I-A/I-E (107614, BioLegend, San Diego, CA, USA), PEcy7-labeled anti-CD206 (141720, BioLegend, San Diego, CA, USA), BV-421 anti-F4/80 (123131, BioLegend, San Diego, CA, USA), and BV605-labeled anti-CCR2 (150615, BioLegend, San Diego, CA, USA). Data were analyzed with FlowJo10.0.5 software (TreeStar Inc., Ashland, OR, USA). PE/Cyanine7 Rat IgG2a κ isotype antibody (400522, BioLegend, San Diego, CA, USA) and APC Rat IgG2b κ isotype antibody (400612, BioLegend, San Diego, CA, USA) were used as controls, as shown in Supplementary Figure S3.

### 2.10. Extraction of Tissue mRNA and Real-Time Polymerase Chain Reaction (RT–PCR)

Total RNA was meticulously extracted from the entire aorta using TRIzol isolation reagent (15596018, Invitrogen; Carlsbad, CA, USA). Subsequently, reverse transcription was conducted on 1 μg of total RNA with the PrimeScript RT Master Mix (RR036A, Takara, Japan). The resulting cDNA served as the template for RT–PCR, employing the SYBR Green Master Mix (RR066A, Takara, Japan) and executed on the CFX manager 96× instrument (Bio–Rad, Hercules, CA, USA). The RT–PCR protocol involved the following: (1) initial denaturation at 95 °C for 3 min, (2) followed by denaturation at 95 °C for 3 s, (3) annealing at 60 °C for 30 min, (4) repeating steps (2) to (3) for an additional 40 cycles, and, finally, (5) melting from 65 °C to 95 °C at 0.5 °C increments. Expression levels of mRNA were normalized to GAPDH, ensuring the accuracy and precision of duplicate measurements. Detailed information regarding the primers utilized for RT–PCR is available in the Key Resources Table.

### 2.11. Bacterial Loads by RT–PCR

Under sterile conditions, total DNA was extracted from blood and aneurysm specimens using the DNeasy Blood & Tissue Kit (69504, Qiagen, Düsseldorf, Germany). To quantify bacterial loads, RT-PCR of 16S rRNA-encoding genes was conducted, following a previously established protocol [16]. In brief, the RT–PCR utilized a universal primer set (338F: ACTCCTACGGGAGGCAGCA and 806R: GGACTACHVGGGTWTCTAAT) with the following reaction conditions: initial denaturation at 95 °C for 3 min, followed by 27 cycles at 95 °C for 30 s, 53 °C for 30 s, and 72 °C for 45 s. Each assay was performed in duplicate with a reaction volume of 20 µL.

### 2.12. IL-1β Measurements

Following the manufacturer’s instructions, plasma IL-1β levels were measured using commercial enzyme-linked immunosorbent assay kits (Invitrogen, Carlsbad, CA, USA). Plasma samples and standard dilution solution were added to the plates and incubated for 90 min at 37 °C. Subsequently, biotin IL-1β antibodies were applied for 60 min at 37 °C, followed by horseradish peroxidase conjugates for 30 min at 37 °C, and chromogenic substrate for 15 min at 37 °C.

### 2.13. Measurement of Fasting Serum Lipids

Mice were subjected to a 12 h fasting period before blood sample collection. Serum was obtained by centrifuging blood samples at 1000× *g* for 10 min. Total cholesterol (TC), triglyceride (TG), high-density lipoprotein (HDL), and the combined sum of very low-density lipoprotein (VLDL) and low-density lipoprotein (LDL) levels were determined using the Cholesterol Quantitation Kit (MAK043, Sigma-Aldrich, St. Louis, MO, USA), Triglyceride Quantitation Kit (MAK266, Sigma-Aldrich, St. Louis, MO, USA), and HDL and LDL/VLDL Quantitation Kit (MAK045, Sigma-Aldrich, St. Louis, MO, USA), following the manufacturer’s instructions.

### 2.14. Intestinal Permeability Assay

Intestinal permeability was assessed following established protocols [17]. Mice were administered FITC-dextran (500 mg/kg body weight) (60842-46-8, Sigma-Aldrich, St. Louis, MO, USA) via oral gavage 5 h prior to euthanasia. The fluorescence of collected samples was measured at an excitation wavelength of 485 nm and emission wavelength of 528 nm using an EnSpire Multimode Plate Reader (PerkinElmer; Shanghai, China). Concentrations of FITC–dextran in the samples were determined by reference to a standard curve.

### 2.15. Bacterial Cultivation

Akkermansia muciniphila (*A. muciniphila*) CICC 24917 were inoculated in GAM medium (Hangzhou Microbial Reagents Co., Ltd., Hangzhou, China). After growing, aliquots from the medium were inoculated on agar medium to pick pure and single colonies. The single and pure colonies were pre-cultured into the medium for growth until OD600 reached 0.6~0.8. Then, 100 μL of each pure bacterial suspension was cultured on 10 mL GAM medium at 37 °C in anaerobic condition until OD600 reached 0.6~0.8. GAM (/L) contained 10 g pancreatic casein peptone, 3 g soy protein, 5 g yeast extract, 2 g beef extract, 13.5 g digested serum, 1.2 g beef liver extract, 2.5 g potassium dihydrogen phosphate, 3 g sodium chloride, 0.3 g cysteine-HCl, 0.15 g sodium thioglycolate, 2 g glucose, 0.1 g porcine gastric mucin, and 0.3 g soluble starch. The pH was adjusted to 7.3 ± 0.1. For bacterial suspension preparation, 100 μL of the cultured GAM medium was added to fresh GAM medium, and the bacterial count was monitored every 6 h until reaching a concentration of 10^9 cells/mL, determined by hemocytometer. The suspension of *A. muciniphila* was washed with PBS, concentrated in anaerobic PBS containing 25% glycerol to a concentration of about 1 × 10^10^ CFU/mL under strict anaerobic conditions, and stored at −80 °C until use. The supernatant of the suspension was preserved as the bacterial secretion [18].

### 2.16. Administration of Akkermansia in Mice

The oral gavage of Akkermansia in mice was performed in accordance with the methods described in the previous literature, confirming that it significantly increased the abundance of this bacterium in the gut [19]. Mice aged 6 weeks were treated by oral gavage with 200 μL of anaerobic PBS, *A. muciniphila* (2 × 10^8^ CFU) (CICC; Beijing, China), heat-killed *A. muciniphila* (2 × 10^8^ CFU), or secretion from *A. muciniphila*. This oral administration was conducted five times a week for a duration of 2 weeks prior to inducing AAA using elastase, followed by an additional 2 weeks post-AAA induction [18].

### 2.17. Endotoxin Detection

Following the manufacturer’s guidelines, plasma lipopolysaccharide (LPS) levels were assessed using a kinetic chromogenic Limulus amebocyte lysate assay (Lonza; Basel, Switzerland) in pyrogen-free tips, test tubes, and Eppendorf tubes. Plasma samples were diluted in pyrogen-free water and then inactivated for 15 min at 90 °C prior to analysis.

### 2.18. DNA Extraction, 16S rRNA Gene Amplification, and Pyrosequencing

Samples were obtained from the contents of the small intestine and colon in mice. Following rapid freezing in liquid nitrogen, the contents were stored at −80 °C. DNA extractions from mouse samples were conducted using the DNeasy PowerSoil Kit, following the provided instructions.

Segments of the 16S rRNA genes were amplified via PCR using a nondegenerate universal primer set (338F and 806R). Unique 7 bp barcodes tailored to each sample were incorporated into the primers, facilitating multiplex sequencing. Subsequently, the resulting amplicons underwent sequencing (250 bps paired-end) at Personal Biotechnology Co., Ltd. (Shanghai, China), leveraging the Illumina MiSeq platform (Illumina, San Diego, CA, USA) in strict adherence to the manufacturer’s protocol.

Microbiome bioinformatic analysis was executed using QIIME2 2019.4, with minor adjustments following the official tutorials. In summary, the raw sequence data underwent demultiplexing using the demux plugin, and subsequent primer trimming was carried out with the cut adapt plugin. Quality control, denoising, merging, and chimera detection steps were accomplished using DADA2 [20]. Nonsingleton amplicon sequence variants (ASVs) were aligned using mafft, and a phylogeny was constructed with fasttree2 [21]. Bacterial taxonomy was assigned based on the SILVA database release 132. The total number of denoised reads included in the analysis amounted to 29,316 sequences.

Sequence data analyses were predominantly conducted using QIIME2 and R packages (v4.0.2). ASV-level α diversity indices, including the Chao1 richness estimator, Shannon diversity index, Simpson index, and Good’s coverage, were computed using the ASV table in QIIME2. Beta diversity analysis was employed to explore structural variations in microbial communities across samples utilizing Bray–Curtis metrics and UniFrac distance metrics, and these were visualized through principal coordinate analysis (PCoA) [22,23]. The significance of differentiation in microbiota structure among groups was evaluated using PERMANOVA or ANOSIM [24,25]. Random forest analysis was utilized to discriminate samples from different groups using QIIME2 with default settings. Linear discriminant analysis effect size (LEfSe) was applied to detect differentially abundant taxa across groups with default parameters. Taxon abundances at the ASV level were statistically compared between inulin diet- and chow diet-fed mice, and between inulin diet- and cellulose diet-fed mice, using MetagenomeSeq, and visualized as Manhattan plots [26].

### 2.19. Statistical Analysis

Statistical analyses were conducted using GraphPad Prism (8.5.0) software, with the exception of microbiota analysis. Normality tests were performed using the Kolmogorov–Smirnov and Shapiro–Wilk tests. Data were presented as the mean ± standard error of the mean (SEM) for normally distributed data. Two-group experiments were analyzed using a two-tailed unpaired Student’s *t* test. Experiments involving four groups were analyzed using one-way ANOVA and Kruskal–Wallis tests, followed by Dunnett’s multiple comparisons test or Tukey’s multiple comparisons test. Non-normally distributed data were represented using box-and-whisker plots and analyzed with the nonparametric Mann–Whitney U test. A *p*-value of less than 0.05 was considered statistically significant.

## 3. Results

### 3.1. Inulin Diet Attenuates Elastase-Induced AAA

**Figure 1:**
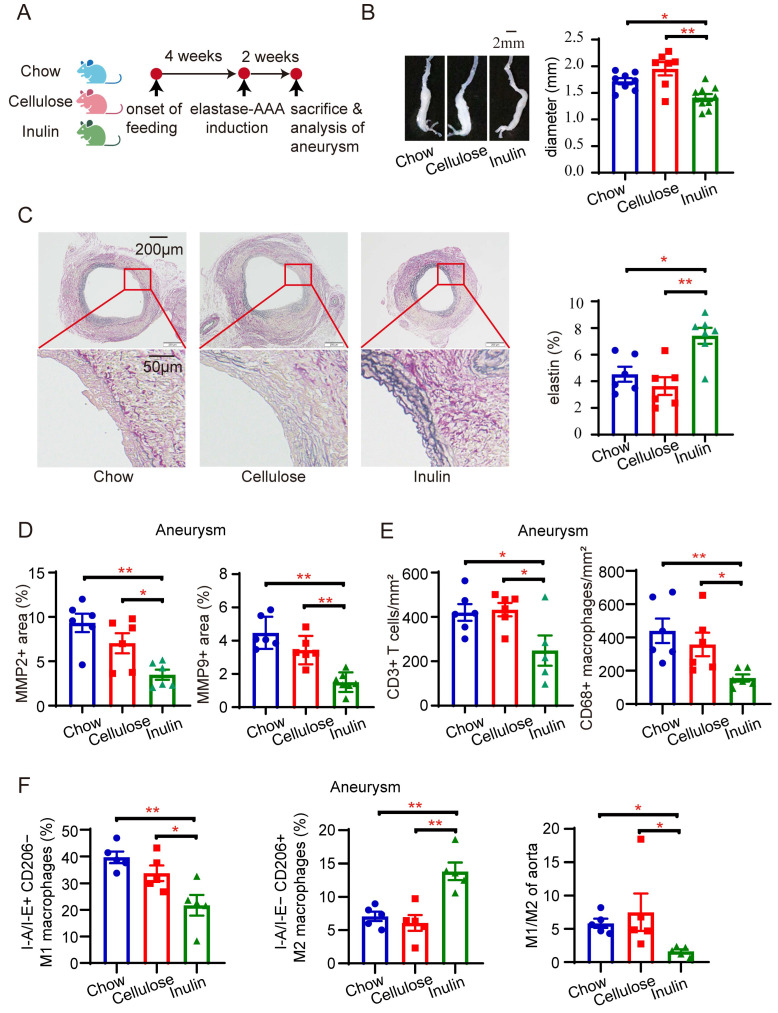
Inulin diet mitigates the progression of elastase-induced abdominal aortic aneurysms in mice. (**A**) Experimental design: SPF C57BL/6 mice were subjected to a 4-week administration of either chow diet, 15% cellulose diet, or 15% inulin diet before AAA induction through elastase injection. The mice were euthanized for assessment two weeks after AAA induction. (**B**) Illustration of aneurysms with representative images and statistical analysis of maximum external diameters from mice on different diets (n = 7–10). (**C**) Representative EVG staining images of aneurysms and quantification of the percentage of elastin area to total aortic area from mice administered different diets (n = 6–7). (**D**) Summary data of immunohistochemical (IHC) staining of the aneurysms, indicating the percentage of MMP2 and MMP9 positive area to the total aortic area (n = 5–7). (**E**) Summary data of IHC staining of the aneurysms showing the number of CD3+ T cells and CD68+ macrophages per mm^2^ of the aorta (n = 5–7). (**F**) Summary flow cytometry data illustrating the percentage of I-A/I-E+ CD206− M1 macrophages, the percentage of I-A/I-E− CD206+ M2 macrophages, and the ratio of M1/M2 macrophages in aneurysms (n = 5). * *p* < 0.05, ** *p* < 0.01, one-way ANOVA followed by Tukey’s multiple comparisons test or Kruskal–Wallis followed by Dunnett’s multiple comparisons test. Scale bars are depicted as indicated in the images. Each dot represents the mean value of 4−8 consecutive sections of an individual mouse in (**C**–**E**).

Prior investigations have assessed the impact of high-fiber diets on disease models by supplementing them with purified fiber components, such as highly fermentable inulin and poorly fermentable cellulose [14]. In this current study, we customized the dietary regimen for mice, incorporating a chow diet (5% cellulose), cellulose diet (15% cellulose), and inulin diet (15% inulin) (Supplementary Table S1). The aim was to explore the potential of high-fiber diets in mitigating the severity of mouse AAAs. Following a 4-week administration period, AAA was induced using elastase, with an additional 2-week dietary administration until euthanasia (Figure 1A).

Our findings indicate that mice receiving an inulin diet exhibited significantly less severe AAA compared with those on chow or cellulose diets. To assess AAA severity, we measured the maximal outer diameter of the aneurysm ex vivo, revealing that aneurysms in mice fed an inulin diet were notably smaller than those in the chow or cellulose diet groups. Importantly, there was no significant difference between the chow and cellulose diets (Figure 1B). EVG staining consistently showed less medial elastin degradation in AAA mice on the inulin diet than in those on the chow or cellulose diets (Figure 1C). Given the crucial roles of MMP2 and MMP9 in elastin degradation, we further assessed their expression through immunohistochemical staining. The results demonstrated that the inulin diet led to a reduction in the positive areas for MMP2 and MMP9 in the AAA lesion (Figure 1D, Supplementary Figure S1A,B). Moreover, AAA lesions from mice on the inulin diet contained fewer CD3+ T cells and CD68+ macrophages than those on the chow or cellulose diets, as shown by immunohistochemical staining (Figure 1E, Supplementary Figure S2A,B). Notably, flow cytometry analyses of aneurysms revealed a decrease in the percentage of classically activated M1 macrophages (I-A/I-E+ CD206−) and an increase in alternatively activated M2 macrophages (I-A/I-E− CD206+) in AAA mice fed the inulin diet compared with those on the chow or cellulose diets (Figure 1F, Supplementary Figure S3). Moreover, lipid profiles among mice on the three diets were similar (Supplementary Figure S4), suggesting that the protective effects of inulin on AAA are independent of lipid metabolism.

### 3.2. High-Fiber Diets Modulate the Microbiota Composition in the Small Intestine and Colon of AAA Mice

Previous studies have highlighted the shift from a chow diet to a refined diet lacking fermentable fiber as a significant factor contributing to differences in the microbiota community [27]. Inulin, a fermentable fiber, has been reported to impact Bacteroides under physiological conditions [28], while promoting the enrichment of Bifidobacteria and Akkermansia in obese or high-fat diet-fed mice [14]. To investigate the effects of high-fiber diets on the microbiota in AAA, we conducted a comprehensive analysis of the small intestine and colon contents in AAA mice fed three different diets using 16S rRNA sequencing two weeks after AAA induction.

**Figure 2:**
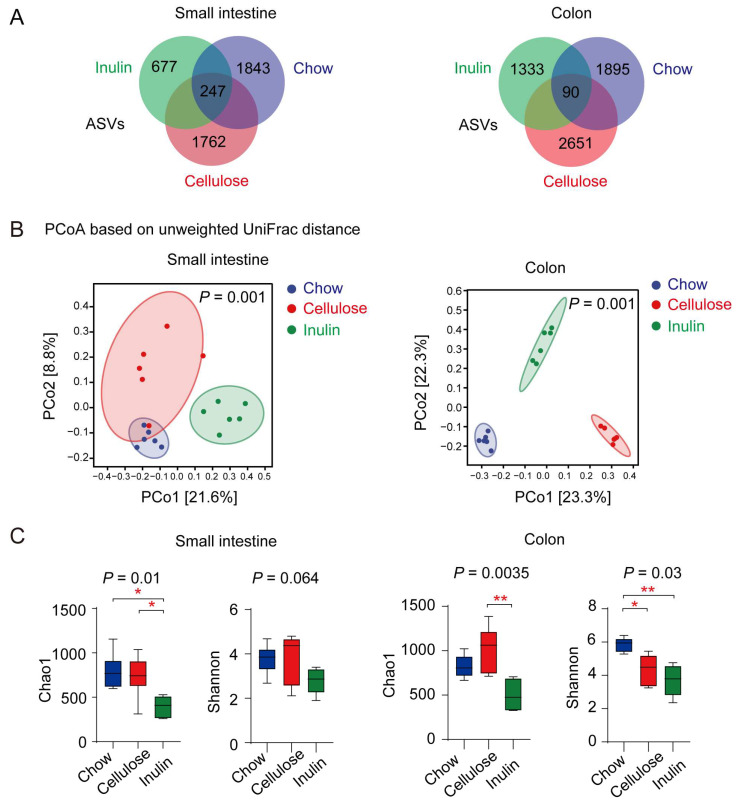
High-fiber diet regulates the microbiota composition in both the small intestine and colon of AAA mice. (**A**) Venn diagrams showing the numbers of unique and shared ASVs of AAA mice with different diets in the small intestine and colon. (**B**) PCoA analyses of β diversity, based on weighted UniFrac distances, showcase significant variations in microbiota profiles in both the small intestine (PERMANOVA, *p* = 0.001) and colon (PERMANOVA, *p* = 0.001) (n = 6). (**C**) Comparisons of α diversity among different diets in the small intestine and colon of AAA mice, evaluated by species richness (Chao1) and microbial diversity (Shannon) at the genus level. * *p* < 0.05, ** *p* < 0.01, with the 95% confidence ellipses displayed.

Rarefaction curves derived from the Shannon index indicated the adequate sequencing depth achieved (Supplementary Figure S5). Venn diagrams illustrated the distribution of unique ASVs in the small intestine and colon of AAA mice subjected to chow, cellulose, and inulin diets. Specifically, 1843, 1762, and 677 unique ASVs were observed in the small intestine, while 1895, 2651, and 1333 unique ASVs were identified in the colon for mice on chow, cellulose, and inulin diets, respectively. Notably, 247 ASVs were shared by all three dietary groups in the small intestine, whereas 90 ASVs were shared in the colon (Figure 2A).

PCoA, utilizing weighted Unifrac distances and Bray–Curtis distances, were employed to evaluate β diversity among distinct groups at the genus level. The results revealed that microbiota profiles in both the small intestine and colon underwent alterations with both inulin and cellulose diets (Figure 2B, Supplementary Figure S6). Notably, regardless of the dietary type, microbiota profiles in the small intestine exhibited distinctions from those in the colon (Supplementary Figure S7).

Alpha diversity indexes, including chao1 and Good’s coverage, were utilized to assess community richness and coverage. Our findings revealed a reduction in richness and an increase in coverage in the small intestine of AAA mice fed an inulin diet (Figure 2C, Supplementary Figure S8). However, community diversity indexes Shannon and Simpson in the small intestine did not exhibit significant differences (Figure 2C, Supplementary Figure S8). In the colon, both inulin and cellulose diets, compared with the chow diet, led to a decrease in community Shannon and Simpson diversity without impacting community richness and coverage (Figure 2C, Supplementary Figure S8).

### 3.3. Inulin Diet Promotes the Enrichment of Akkermansia in Both the Small Intestine and Colon of AAA Mice

**Figure 3:**
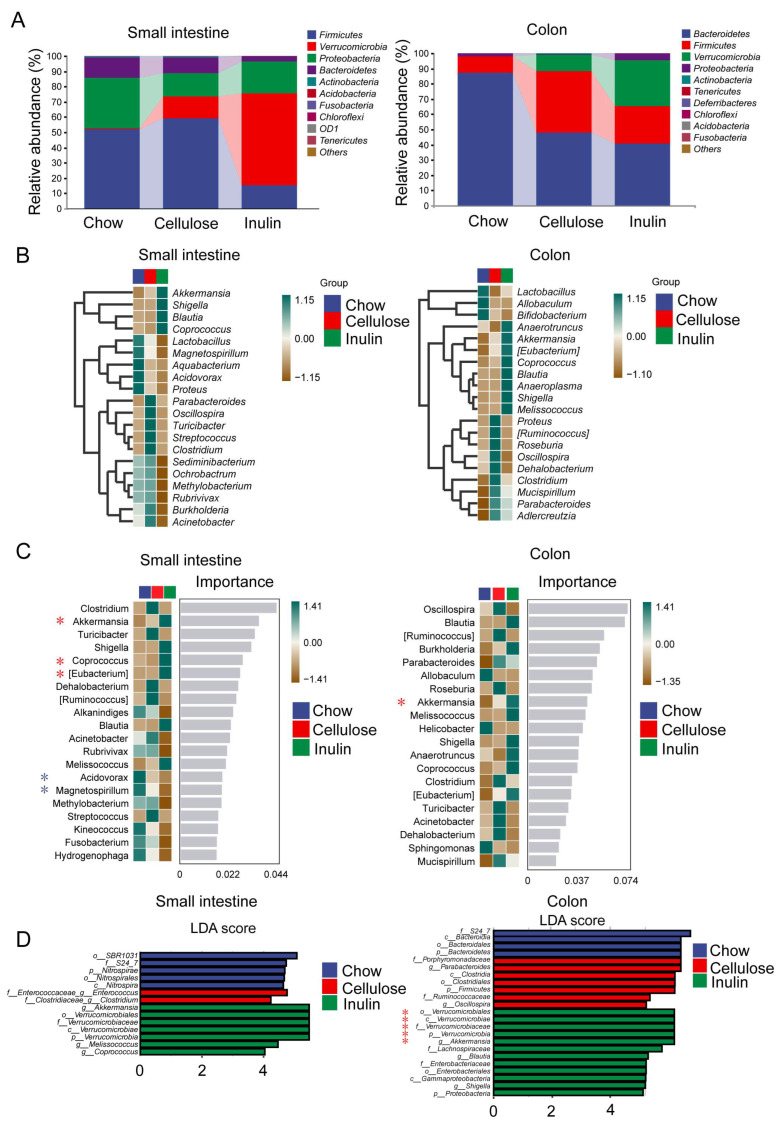
Dietary inulin increases Akkermansia abundance in the small intestine and colon of AAA mice. (**A**) Microbiota profiles at the phylum level in the contents of the small intestine and colon. (**B**) UPGMA clustering based on Euclidean distances and Pearson correlation coefficients of the microbiota in small intestine and colon contents. Random forest analysis (**C**) and LEfSe analysis (**D**) of the microbiota in small intestine and colon contents. (**D**) The length indicates the effect size associated with each taxon. * *p* < 0.05 by Wilcoxon signed-rank test; all-against-all strategy; LDA score > 4. (**A**–**D**), n = 6.

The Verrucomicrobia phylum, which only comprises the genus Akkermansia, was enriched by the inulin diet in both the small intestine and colon (Figure 3A). Moreover, the Firmicutes to Bacteroidetes ratio remained unaffected by the inulin diet in both the small intestine and the colon. The analysis at the genus level, including the heatmaps of relative abundance (Supplementary Figure S9), unweighted pair group method with arithmetic mean clustering (Figure 3B), and unsupervised clustering based on the random forest algorithm (Figure 3C), collectively suggested that the inulin diet specifically enhanced Akkermansia populations in the small intestine and colon of AAA mice. Within the microbiota enriched by the inulin diet, Akkermansia had the highest importance ranking in the small intestine and the third highest in the colon. Additionally, the inulin diet elevated the levels of SCFA-producing Coprococcus and Eubacterium, while reducing nonfermenting Acidovorax and Magnetospirillum in the small intestine (Figure 3C).

In order to illustrate the differences at each taxonomic level, we created histograms of linear discriminant analysis (LDA) (Figure 3D) based on LDA effect size (LEfSe) analysis. The outcomes highlighted that Akkermansia, along with its phylum, class, order, and family, played a pivotal role in both the small intestine and colon of AAA mice subjected to the inulin diet. LDA aimed at reducing potential biases related to rarefaction, and consistently identified Akkermansia as the primary responder to inulin in both the small intestine and the colon. (Supplementary Figures S10 and S11).

### 3.4. Inulin’s Impact on Intestinal Barrier of AAA Mice

**Figure 4:**
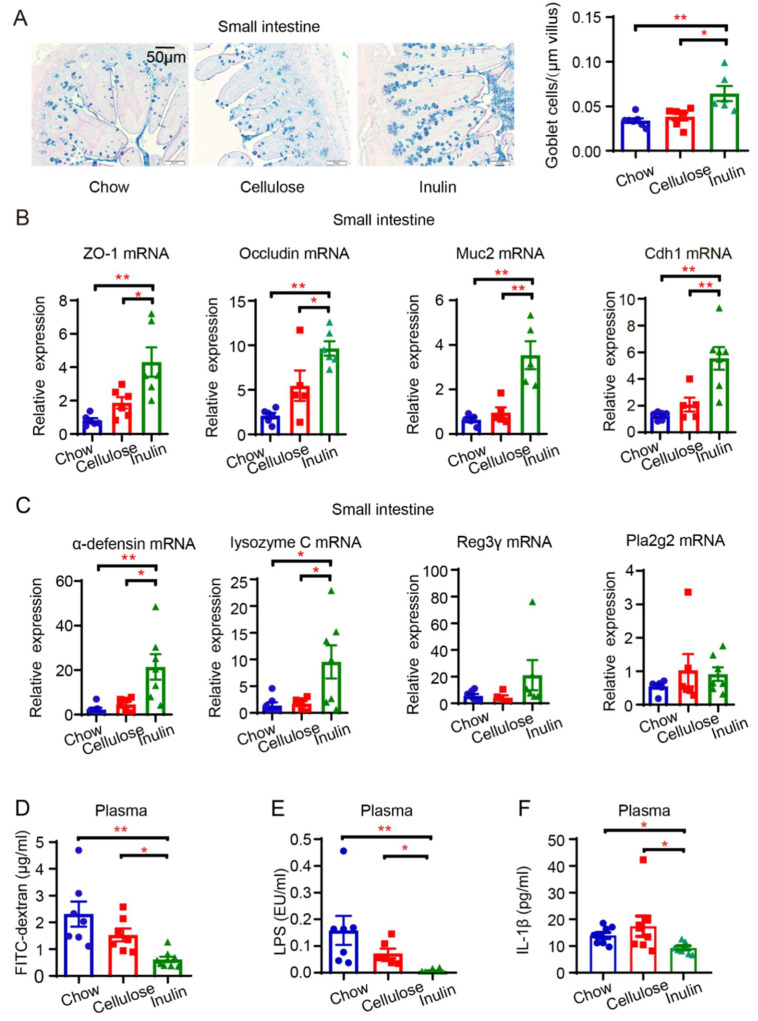
Inulin diet enhances intestinal barrier in AAA mice. (**A**) Representative PAS-AB staining of the small intestine and quantification of GCs per µm of villus (n = 6–7). (**B**) Relative expression of physical barrier-associated genes including ZO-1, Occludin, Muc2, and Cdh1 in the small intestine (n = 5–7). (**C**) Relative expression of chemical barrier-associated genes including α-defensin, lysozyme C, RegIIIγ, and Pla2g2 in the small intestine (n = 5–7). (**D**) Measurement of plasma FITC fluorescence intensity in AAA mice with different diets 6 h after gavage with FITC-dextran (n = 7–9). (**E**) Quantification of plasma LPS concentration using chromogenic endpoint Tachypleus assay in AAA mice (n = 6–7). (**F**) Measurement of plasma IL-1β concentration by ELISA (n = 8). Scale bar is depicted as indicated in the images. * *p* < 0.05, ** *p* < 0.01. One-way ANOVA followed by Tukey’s multiple comparisons test or Kruskal–Wallis followed by Dunnett’s multiple comparisons test.

As previously documented, the administration of inulin and Akkermansia has been reported to restore the intestinal barrier [29,30]. Consequently, we systematically evaluated the impact of inulin on the intestinal barrier in AAA mice. Utilizing PAS-AB staining, we observed a notable increase in the number of GCs in the small intestine of AAA mice subjected to the inulin diet (Figure 4A). However, the number of colonic GCs did not exhibit a significant increase (Supplementary Figure S12A). Subsequently, we assessed the gene expression of key tight junction proteins, including zonula occludens-1 (ZO-1), occludin, gel-forming protein organizing the mucus layer mucin2 (Muc2), and cell adhesion protein cadherin1 (Cdh1), to evaluate the integrity of the physical barrier in both the small intestine and the colon. Inulin demonstrated a notable impact on enhancing the integrity of the physical barriers in the small intestine, but showed no significant effect on the colon (Figure 4B, Supplementary Figure S12B). The intestinal mucosa plays a pivotal role in maintaining the gut’s chemical barrier through the secretion of antimicrobial peptides such as α-defensin, lysozyme C, C-type lectin (Reg3γ), and phospholipase A2 group-II (Pla2g2) [31,32]. The inulin diet substantially augmented the gene expression of antimicrobial peptides in AAA mice, particularly α-defensin and lysozyme C in the small intestine and lysozyme C and pla2g2 in the colon, while cellulose failed to induce a significant improvement in the chemical barrier (Figure 4C, Supplementary Figure S12C). Moreover, we assessed intestinal permeability through FITC-dextran gavage. The plasma FITC fluorescence intensity in AAA mice on the inulin diet was significantly lower than that observed in mice on the chow or cellulose diet (Figure 4D). This illustrates that inulin effectively reduces intestinal permeability, primarily by enhancing the integrity of the intestinal barrier, particularly in the small intestine.

Upon compromise of the integrity of the intestinal barrier, pathogen-associated molecular patterns gain access to the portal circulation, reaching distant sites and triggering inflammatory responses [33]. Our observations revealed a significant reduction in plasma LPS levels (Figure 4E) and the systemic pro-inflammatory cytokine IL-1β (Figure 4F) in AAA mice on the inulin diet. Additionally, positive correlations were identified among intestinal permeability, LPS, and AAA diameter (Supplementary Figure S13). These findings collectively indicate that the reinforced intestinal barrier in AAA mice fed the inulin diet decreased the translocation of LPS, which was positively correlated with the severity of AAA.

### 3.5. The Inulin Diet Decreases Ly6Chi Monocytes in the Bone Marrow, Blood, and Infiltration into the Aneurysm

**Figure 5:**
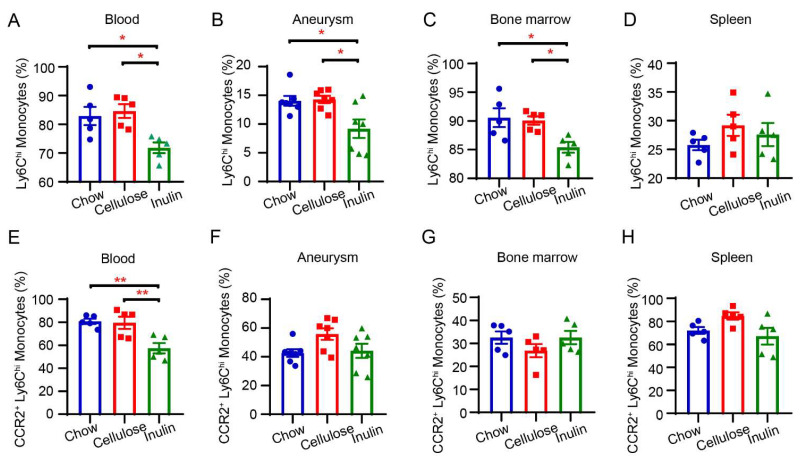
Inulin diet decreases Ly6Chi monocytes in bone marrow, peripheral blood, and aneurysms. The percentages of Ly6Chi monocytes (of Ly6G- CD11b+ monocytes) in the blood (**A**), aneurysm (**B**), bone marrow (**C**), and spleen (**D**) were measured by flow cytometry (n = 5–7). The percentages of CCR2+ Ly6Chi monocytes (of Ly6Chi monocytes) in the blood (**E**), aneurysm (**F**), bone marrow (**G**), and spleen (**H**) were measured by flow cytometry (n = 5–7). * *p* < 0.05, ** *p* < 0.01. One-way ANOVA followed by Tukey’s or Dunnett’s multiple comparisons test.

Translocated bacterial component LPS serves as a potent stimulator of innate immunity. We utilized flow cytometry to detect monocytes and neutrophils in the peripheral blood of AAA mice (Supplementary Figure S14A). The inulin diet did not alter the proportions of CD11b+ Ly6G+ neutrophils or CD11b+ Ly6Clow patrolling monocytes in the blood but reduced pro-inflammatory CD11b+ Ly6Chi monocytes both in the peripheral blood and aneurysms of AAA mice (Figure 5A,B, Supplementary Figure S14B). The percentage of Ly6Chi monocytes also decreased in the bone marrow but not in the spleen of AAA mice with inulin diet (Figure 5C,D). CCR2 plays a significant role in the recruitment of pro-inflammatory Ly6Chi monocytes to AAA lesions [34]. We also analyzed CCR2 expression on Ly6Chi monocytes in the aneurysm, blood, bone marrow, and spleen. In AAA mice fed the inulin diet, the CCR2 expression of Ly6Chi monocytes from the peripheral blood reduced notably (Figure 5E), yet remained unaltered in the aneurysm, bone marrow, and spleen (Figure 5F–H).

In summary, we found that inulin decreased pro-inflammatory Ly6Chi monocytes in the peripheral blood, aneurysm, and bone marrow and CCR2 expression on Ly6Chi monocytes from the peripheral blood.

### 3.6. Administration of Akkermansia Muciniphila Ameliorates AAA in Mice by Enhancing the Intestinal Barrier

As previously reported, the administration of *A. muciniphila* increased the endocannabinoid levels in the intestine that regulate inflammation and antimicrobial peptide secretion, thereby improving the intestinal barrier in diet-induced obesity [29,30]. Therefore, we hypothesized that *A. muciniphila* could enhance the intestinal barrier, attenuate bacterial components’ translocation, and, consequently, alleviate AAA.

**Figure 6:**
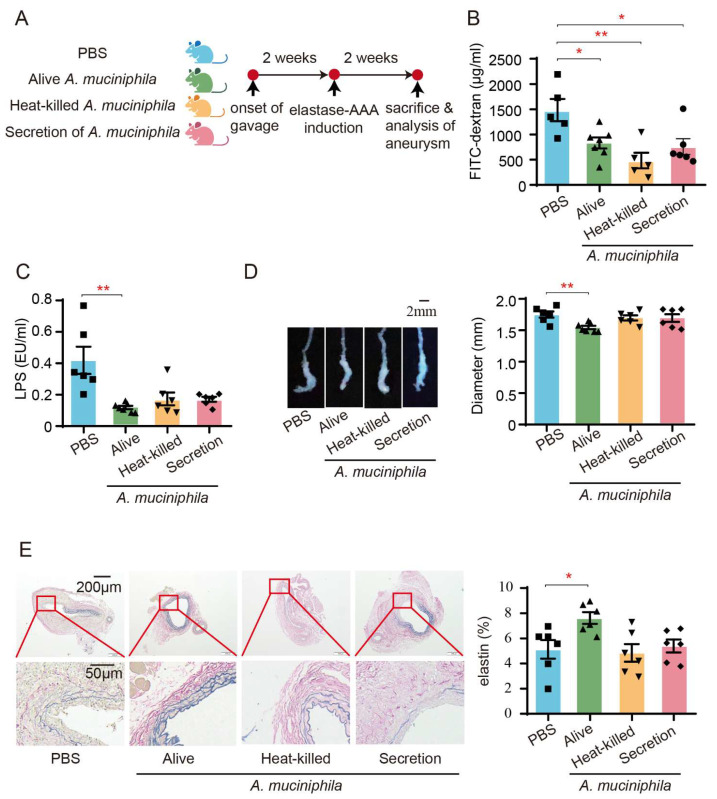
Administration of *A. muciniphila* improves intestinal barrier and mitigates AAA in mice. (**A**) Study overview: SPF C57BL/6J mice were administered *A. muciniphila* by gavage every 5 days per week for 2 weeks prior to the induction of AAA using elastase. The administration of *A. muciniphila* continued for two weeks after the induction of AAA. The mice were then euthanized for evaluation. (**B**) The plasma FITC fluorescence intensity of AAA mice with or without administration of *A. muciniphila* was measured 6 h after gavage with FITC-dextran (n = 5–7). (**C**) The plasma concentrations of LPS were measured by chromogenic endpoint Tachypleus assay (n = 6–7). (**D**) Representative images of aneurysms and statistical analysis of maximum external diameter from mice with or without administration of *A. muciniphila* (n = 6–7). (**E**) Representative EVG staining images of aneurysms and quantification of the percentages of elastin area to total aortic area from mice with or without administration of *A. muciniphila* (n = 6). Scale bars are depicted as indicated in the images. Each dot represents the mean value of 4 consecutive sections of an individual mouse. * *p* < 0.05, ** *p* < 0.01. One-way ANOVA followed by Dunnett’s multiple comparisons test.

To evaluate the influence of *A. muciniphila* on AAA-associated intestinal barrier dysfunction, we administered *A. muciniphila* to AAA mice via oral gavage (Figure 6A). The results showed that the AAA mice with an administration of live *A. muciniphila* exhibited reduced plasma FITC fluorescence intensity, systemic LPS levels, aneurysm diameter, and elastin degradation (Figure 6B–E). Conversely, treatment with heat-killed *A. muciniphila* and *A. muciniphila* secretions did not impact aneurysm diameter, elastin degradation, or circulating endotoxin levels in AAA mice, despite demonstrating improvements in intestinal permeability (Figure 6B–E).

## 4. Discussion

**Figure 7:**
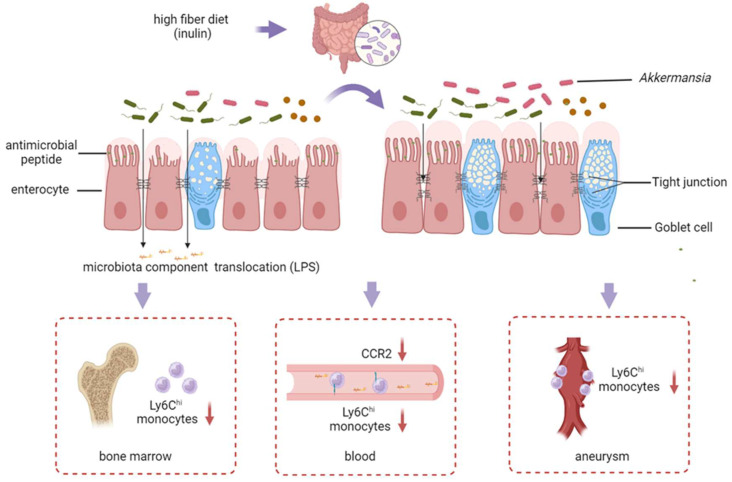
Take home figure. The inulin diet increased Akkermansia levels, contributed to the restoration of the intestinal barrier, and decreased systemic LPS in AAA mice. The inulin diet also led to a reduction in pro-inflammatory Ly6Chi monocytes in the bone marrow, circulating blood, and the aneurysm. Both the inulin diet and Akkermansia mitigated AAA severity in mice. Image created by Biorender, with permission.

This study delved into the effects and mechanisms by which a high-fiber diet suppresses AAA progression. The highly fermentable inulin diet demonstrated protective roles in elastase-induced AAA mice by restraining elastin degradation and reducing pro-inflammatory cell infiltration, whereas cellulose supplementation did not show similar effects. Akkermansia, identified as the dominant responder to inulin in AAA, emerged as a crucial factor in reinforcing intestinal permeability and alleviating AAA. The enhanced intestinal barrier in AAA mice with an inulin diet limited the translocation of bacteria component LPS, resulting in decreased systemic inflammatory responses mediated by Ly6Chi monocytes and a reduction in bone marrow production, thus leading to diminished infiltration of these monocytes into the aneurysm. The main findings of this study are summarized in Figure 7.

A recent randomized prospective study that integrated omics measurements of the microbiome and host indicated that a high-fiber diet did not alter microbial community diversity, whereas a highly fermented food diet steadily increased microbiota diversity and decreased inflammatory marker levels [35]. The distinguishing factor between inulin and cellulose, fiber fermentability, seems to drive the differences in gut microbiota composition and inflammatory markers. A previous study revealed that inulin’s impact on the gut microbiota promoted intestinal epithelial proliferation and prevented microbiota encroachment into the mucosa, thus protecting against metabolic syndrome in a microbiota-dependent manner, but cellulose did not [14]. Our results correspondingly highlight the important roles of the fermentable fiber inulin in disease protection and elucidate the effects of different high-fiber diets on AAA.

Akkermansia, recognized for its role in the degradation of intestinal content or host-derived mucins, contributes to the production of propionate and acetate, thus enhancing the intestinal mucus layer and providing defense against various diseases, including atherosclerosis, diabetes mellitus, and cancer [30,36]. Our findings strongly indicate that Akkermansia is the primary responder to inulin, demonstrating its crucial role in enhancing intestinal barrier and protecting against AAA. By combining dietary interventions, assessing immune responses, and analyzing the microbiome, we have the potential to offer personalized and population-wide insights for managing AAA.

Previous studies have highlighted the potential beneficial effects of the outer membrane protein Amuc_1100 and a secreted protein P9 from *A. muciniphila*, as they were found to partially replicate the positive impacts of the bacterium [37,38]. Contrary to expectations, our results indicate that neither heat-killed *A. muciniphila* nor its secreted components play a pivotal role in ameliorating AAA. This prompts further exploration to identify the active ingredients and underlying mechanisms responsible for the observed effects, particularly focusing on investigating Amuc_1100. Notably, Amuc_1100 has shown improvements in the intestinal barrier in patients with obesity during a randomized controlled trial [38]. 

In the context of this study, the analysis of α diversity indicated that the inulin diet decreased diversity, a characteristic commonly linked to dysbiosis. Nevertheless, the relationship between α diversity and intestinal homeostasis or disease conditions has shown inconsistency in previous studies [39,40]. Our results are consistent with prior findings that an inulin diet decreases the α diversity of fecal microbiota under conditions like hypertension and high-fat diet-induced mice [14,41]. Importantly, our study suggests that inulin can exert a protective role against diseases, even when α diversity does not increase in response to dietary intervention.

Researches have predominantly focused on alterations in microbial communities both in the small intestine and the colon. The findings in this study, particularly the augmentation of Akkermansia and the reinforcement of the intestinal barrier, emphasize the critical role of the intestine in inflammatory diseases in distal organs. Previous studies have revealed crosstalk between the intestine and distal organs including the liver [42], adipose tissue [43], and brain [44]. This study established the crosstalk between the intestine and the inflamed aorta, and the potential of modulating this crosstalk for therapeutic purposes needs to be explored.

A substantial accumulation of macrophages occurs within aneurysm walls, predominantly derived from circulating monocytes. Pro-inflammatory Ly6Chi monocytes play a more pivotal role in the progression of AAA compared with Ly6Clow monocytes [34]. Notably, our findings indicate that the inulin diet serves as a preventive measure against increased intestinal permeability. This protective effect can, in turn, hinder the generation of Ly6Chi monocytes in the bone marrow, subsequently inhibiting the migration of Ly6Chi monocytes into the aneurysm and contributing to the mitigation of AAA progression.

The conventional treatment approach for AAA involving the administration of oral antibiotics presents several drawbacks. Firstly, oral antibiotics induce a significant disturbance in the microbiota, resulting in diminished abundance, diversity, and evenness, consequently compromising the integrity of the intestinal barrier [45]. Extensive clinical trials have unequivocally shown that antibiotics are not efficacious in treating AAA [46]. In contrast with oral antibiotics, an inulin diet or oral supplementation with live *A. muciniphila* represents a lifestyle-based intervention for inflammatory diseases. The clinical trial has established the safety and well-tolerated nature of daily oral supplementation of live *A. muciniphila* in human volunteers with overweight and obesity [47]. Additionally, a randomized cross-over trial involving 20 participants with overweight demonstrated that dietary inulin supplementation significantly increased the abundance of SCFA-producing gut microbiota compared with a placebo, stabilized blood glucose levels, and improved insulin sensitivity [48]. These findings provide robust clinical evidence for the metabolic and anti-inflammatory benefits of inulin supplementation. However, studies investigating the potential therapeutic application of inulin in AAA remain scarce and warrant further validation.

Given the suppressive impact of the inulin diet on the inflammatory response, caution is advised in administering this intervention to acutely infected patients. Additionally, the potential protective roles of other highly fermentable fibers, such as pectin, β-glucan, and dextrin, in AAA warrant further exploration. Our research was conducted exclusively with male mice, in accordance with data suggesting a higher incidence of AAA in men compared with women, and the heightened susceptibility of male mice across all mouse models [49]. It is important to recognize that this design may limit the applicability of the findings to female mice. Future studies encompassing both male and female mice, with a specific emphasis on gender-related discrepancies, are essential to establish a comprehensive understanding of the impacts of these dietary interventions on AAA.

## 5. Conclusions

This study demonstrates that the fermentable fiber inulin mitigates AAA progression by fortifying the intestinal barrier, possibly attributed to the enrichment of Akkermansia. This finding represents a substantial step forward in the clinical translation of an inulin diet as a non-invasive therapeutic approach for AAA.

## Data Availability

The original contributions presented in the study are included in the article and Supplementary Materials, further inquiries can be directed to the corresponding author.

## Supplementary Materials

The following supporting information can be downloaded at: https://www.mdpi.com/article/10.3390/biomedicines13040920/s1, Figure S1: Inulin diet reduces the expression of MMP2 and MMP9 in the aneurysms of AAA mice; Figure S2: Inulin diet reduces the infiltration of CD3+ T cells and CD68+ macrophages in the aneurysms of AAA mice; Figure S3: Gating strategy for the detection of M1 and M2 macrophages; Figure S4: High-fiber diet has no effect on lipids profile; Figure S5: The rarefaction curve of the microbiota; Figure S6: β diversity of microbiota composition in AAA mice with different diets; Figure S7: β diversity of microbiota from the small intestine and colon of AAA mice; Figure S8: α diversity of the microbiota in the small intestine and colon of AAA mice fed different diets; Figure S9: Heatmap at the genus level with respect to abundance; Figure S10: MetagenomeSeq analyses of the microbiota from the small intestine of AAA mice fed with different diets; Figure S11: MetagenomeSeq analyses of the microbiota from the colon of AAA mice fed with different diets; Figure S12: Effects of the inulin diet on the colon barrier of AAA mice; Figure S13: Pearson correlation analyses between intestinal permeability, plasma LPS, and aneurysm diameter; Figure S14: Inulin diet has no impact on Ly6Clow monocytes and neutrophils in AAA mice; Table S1: The ingredients of diet for mice; Key Resources Tables: Antibody, reagent information, and RT-PCR primers.

## Abbreviations

The following abbreviations are used in this manuscript:

AAA	abdominal aortic aneurysm
MMP	matrix metalloproteinase
LPS	lipopolysaccharide
CCR2	C-C chemokine receptor 2
IL	interleukin
SCFA	short-chain fatty acids
SPF	specific pathogen-free
SEM	standard error of the mean
EVG	Elastica van Gieson
IHC	immunohistochemical
ASV	amplicon sequence variants
PCoA	principal coordinate analyses
LDA	linear discriminant analysis
LEfSe	LDA effect size
PAS-AB	Schiff–Alcian Blue
GCs	goblet cells
ZO-1	zonula occludens-1
Muc2	mucus layer mucin2
Cdh2	cell adhesion protein cadherin1
Reg3γ	C-type lectin
Pla2g2	phospholipase A2 group-II
*A. muciniphila*	Akkermansia muciniphila
