# Supplementary material for: Inulin Diet Alleviates Abdominal Aortic Aneurysm by Increasing Akkermansia and Improving Intestinal Barrier
Source: Biomedicines. 2025 Apr 9;13(4):920. doi: 10.3390/biomedicines13040920 (PMC12024805; doi:10.3390/biomedicines13040920)
Supplement: Supplementary file 1 [file biomedicines-13-00920-s001.zip › biomedicines-3531855-supplementary.pdf]

biomedicines

Supplementary Appendix

Inulin Diet Alleviates Abdominal Aortic Aneurysm by Increasing Akkermansia and Improving Intestinal Barrier

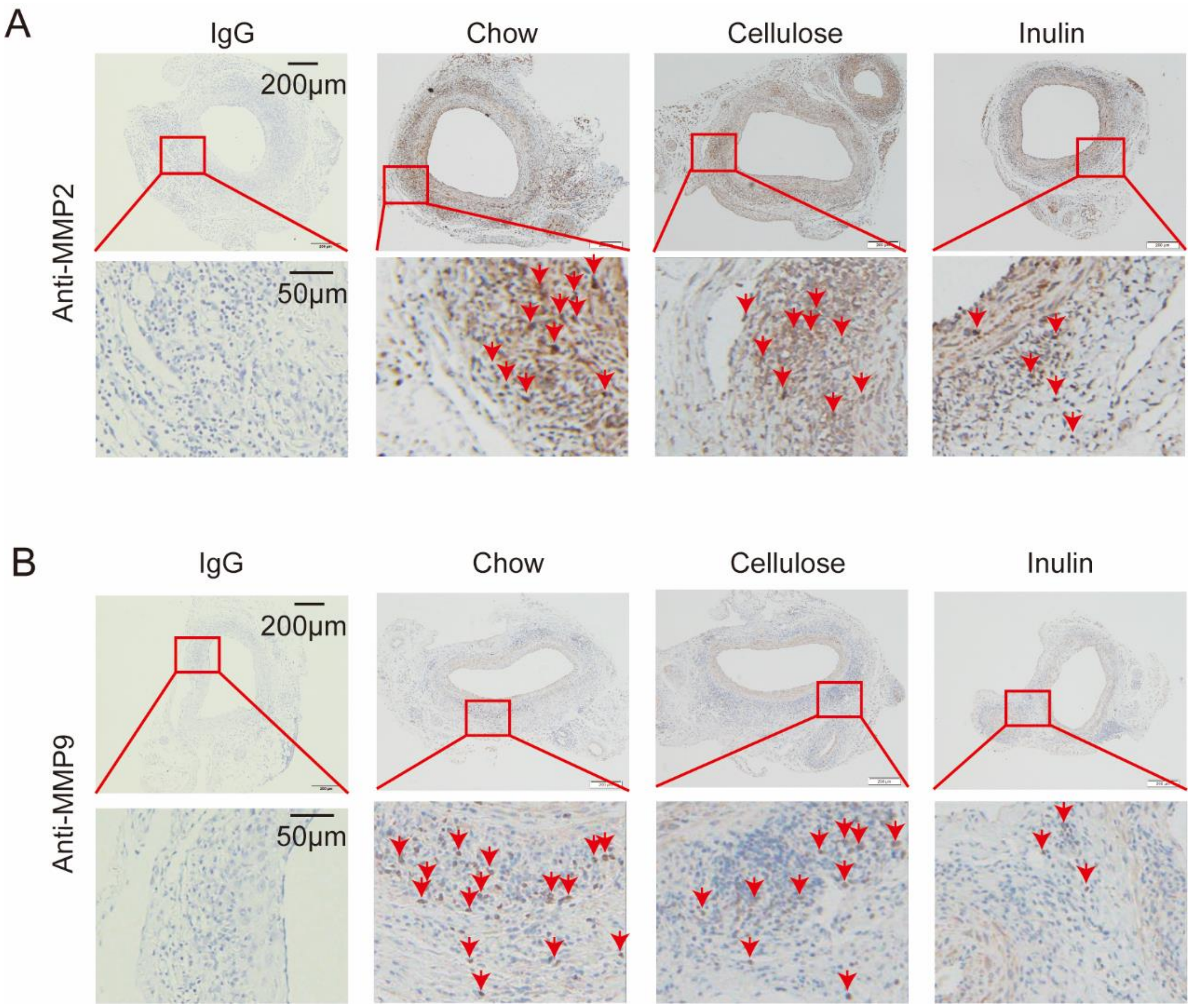

Figure S1. Inulin diet reduces the expression of MMP2 and MMP9 in the aneurysms of AAA mice. Representative immunohistochemistry staining images of aneurysms, subjected to anti-MMP2 **(a)**, anti-MMP9 antibodies **(b)**, or IgG antibody isotype control were obtained from AAA mice exposed to various diets. The scale bars, as indicated in the images, provide a reference for measurement. Notably, positive staining is marked by red arrows, highlighting areas of interest and potential significance in the context of different dietary interventions for AAA.

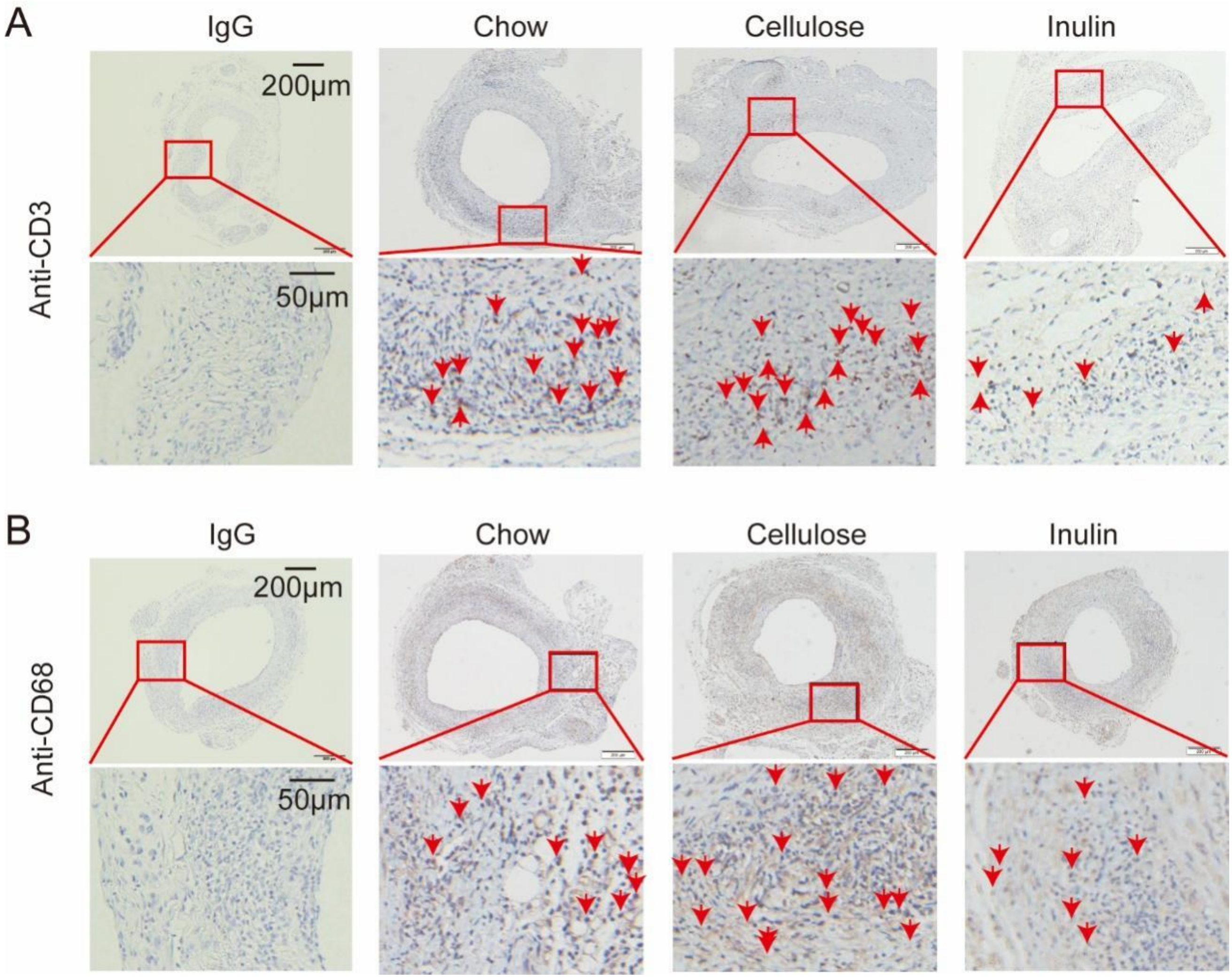

Figure S2. Inulin diet reduces the infiltration of CD3+ T cells and CD68+ macrophages in the aneurysms of AAA mice. Representative immunohistochemistry staining images of aneurysms, utilizing anti-CD3 antibodies **(a)**, anti-CD68 antibodies **(b),** or IgG antibody isotype control, were acquired from AAA mice subjected to distinct dietary regimens. Scale bars, as indicated in the images, serve as reference markers. Positive staining is highlighted by red arrows, signifying areas of interest and potential relevance in the context of varied dietary influences on AAA.

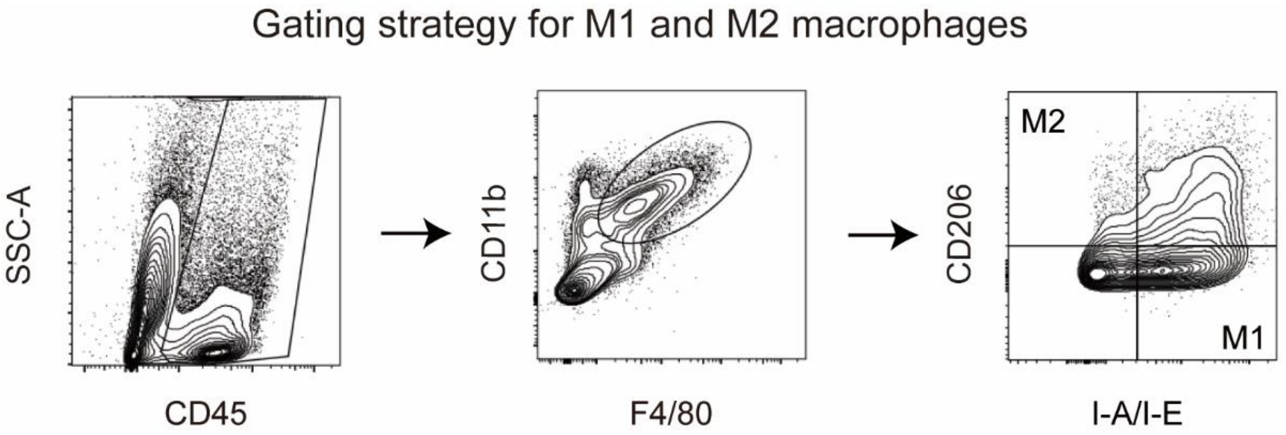

Figure S3. Gating strategy for the detection of M1 and M2 macrophages. The flow cytometry gating strategy for distinguishing I-A/I-E+ C206- M1 and I-A/I-E- C206+ M2 macrophages involved the utilization of isotype antibodies as controls.

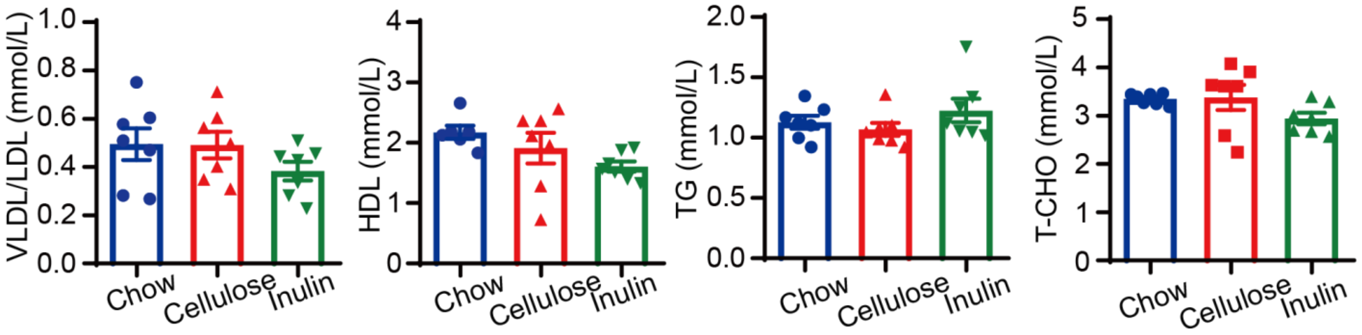

Figure S4. High-fiber diet has no effect on lipids profile. After 6 weeks of diet administration, plasma was collected for lipid analysis. Quantification of plasma lipids, including VLDL/LDL, HDL, triglycerides, and total cholesterol, was performed on samples from a total of 6–7 individuals. Statistical analysis was conducted using one-way ANOVA followed by Tukey's multiple comparisons test for VLDL/LDL, HDL and triglycerides, while total cholesterol data were analyzed using Kruskal-Wallis followed by Dunn's multiple comparisons test. VLDL, very low-density lipoprotein; LDL, low-density lipoprotein-cholesterol; HDL, high-density lipoprotein cholesterol; TG, triglyceride; T-CHO, total cholesterol.

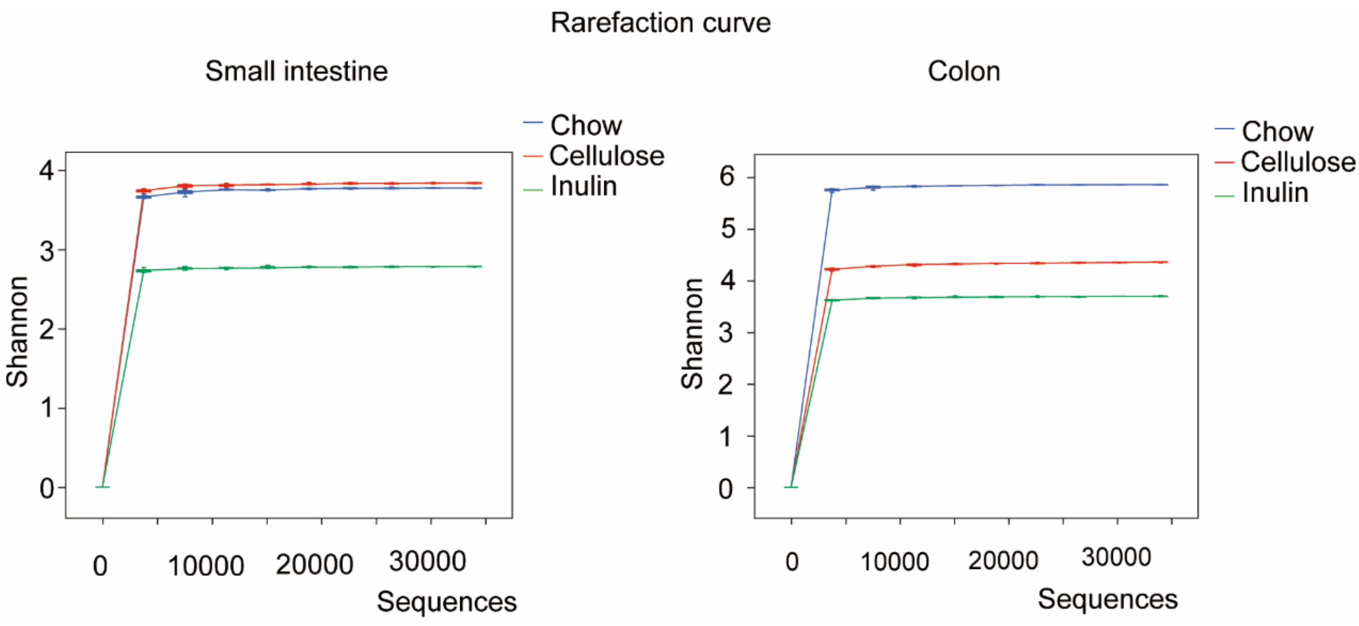

Figure S5. The rarefaction curve of the microbiota. A rarefaction curve based on the Shannon index was generated to assess the diversity of the microbiota in the small intestine and colon of AAA mice fed different diets. (n = 6).

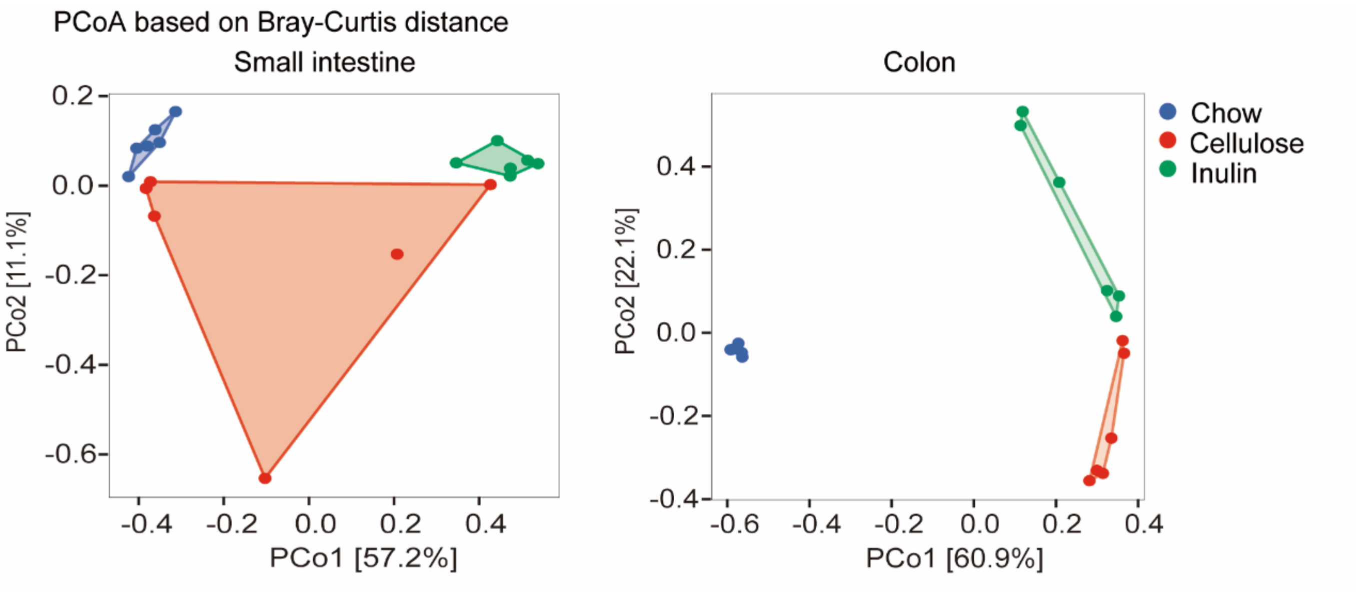

Figure S6. β diversity of microbiota composition in AAA mice with different diets. β diversity was visualized using principal coordinate analysis (PCoA) based on the Bray-Curtis distances of gut microbiota from the small intestine (permutational multivariate analysis of variance (PERMANOVA), *P* = 0.001) and colon (PERMANOVA, *P* = 0.001) of AAA mice fed different diets. (*n* = 6).

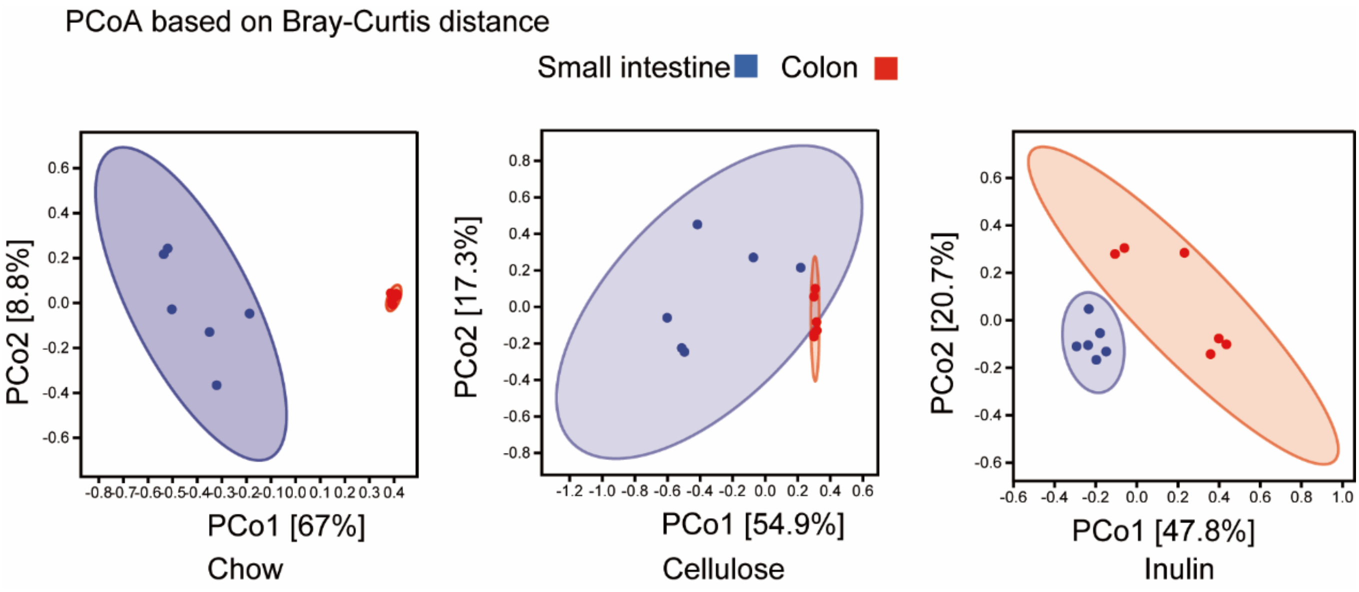

Figure S7. β diversity of microbiota from the small intestine and colon of AAA mice. β diversity was visualized using principal coordinate analysis (PCoA) based on the Bray-Curtis distances of the gut microbiome from the small intestine and colon of AAA mice fed chow diet (PERMANOVA, *P* = 0.007), cellulose diet (PERMANOVA, *P* = 0.001), and inulin diet (PERMANOVA, *P* = 0.003) (*n* = 6). The 95% confidence ellipses were shown in the PCoA plot.

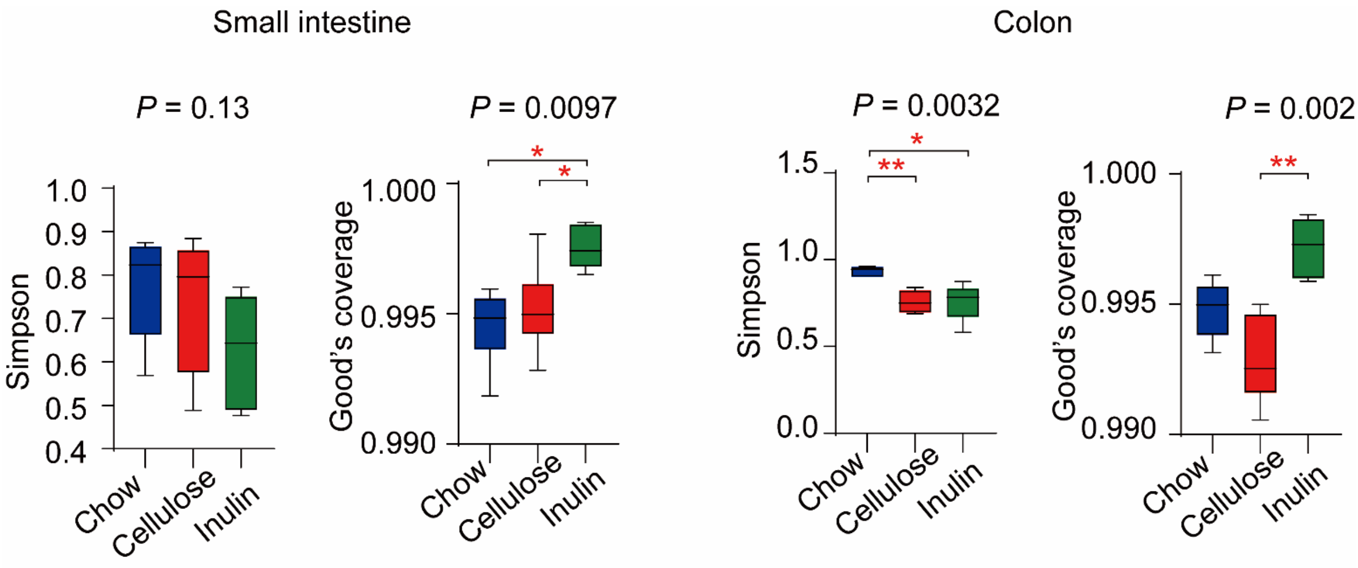

Figure S8. α diversity of the microbiota in the small intestine and colon of AAA mice fed different diets. α diversity of the microbiota in the small intestine and colon of AAA mice fed different diets was visualized using Simpson and Good's coverage indexes ($n = 6$). Significance levels are indicated as * $P < 0.05$ and ** $P < 0.01$. In the boxplots, the bounds of the box represent the first and third quartiles, with the median shown as a thick line in the middle. Whiskers extend to values within 1.5 times the difference between the 25th and 75th percentiles.

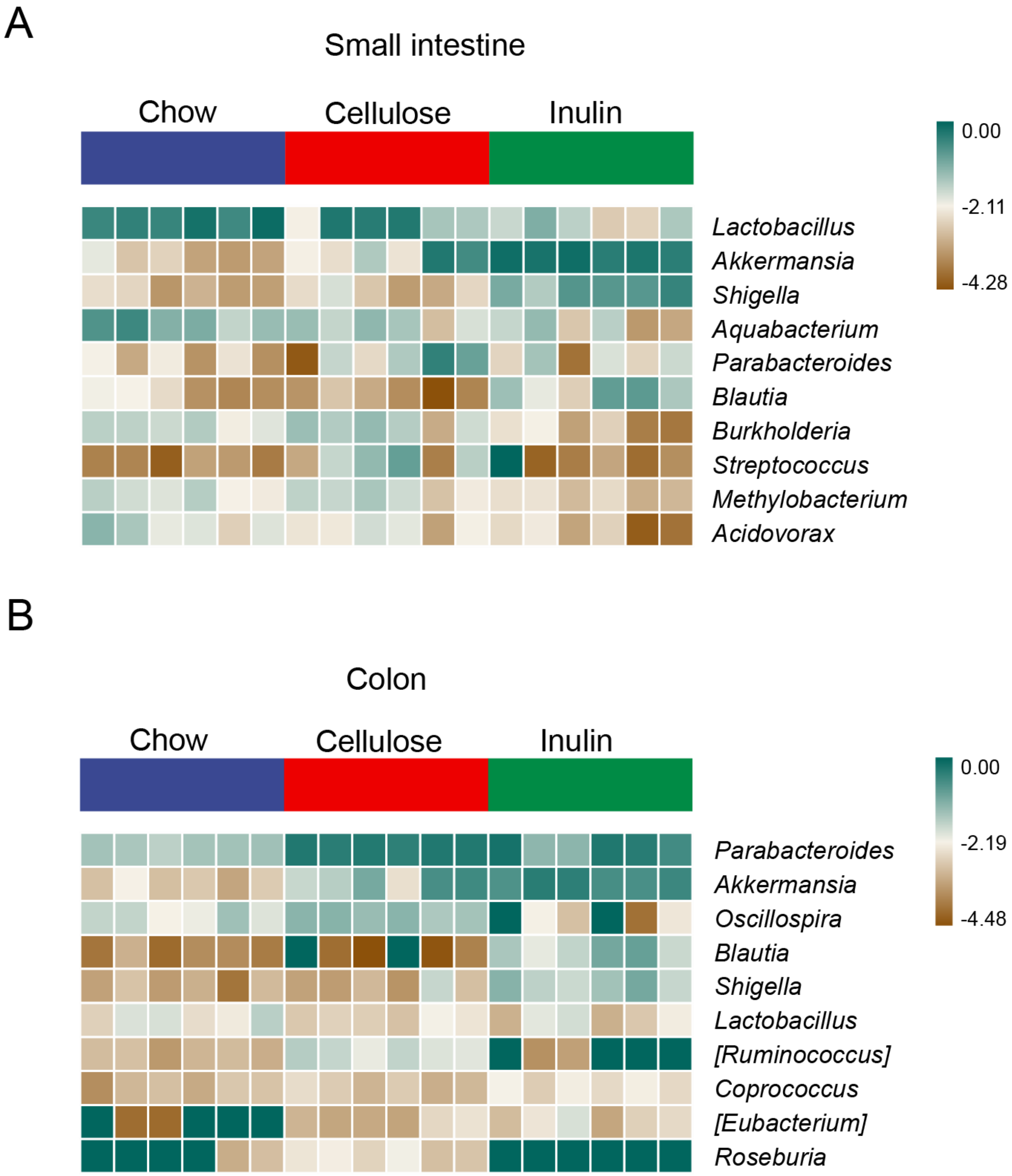

Figure S9. Heatmap at the genus level with respect to abundance. The heatmap illustrates the top 10 dominant bacteria at the genus level based on their abundance in the small intestine **(a)** and colon **(b)**. Relative abundance data are presented, and each column corresponds to a specific specimen. *n* = 6.

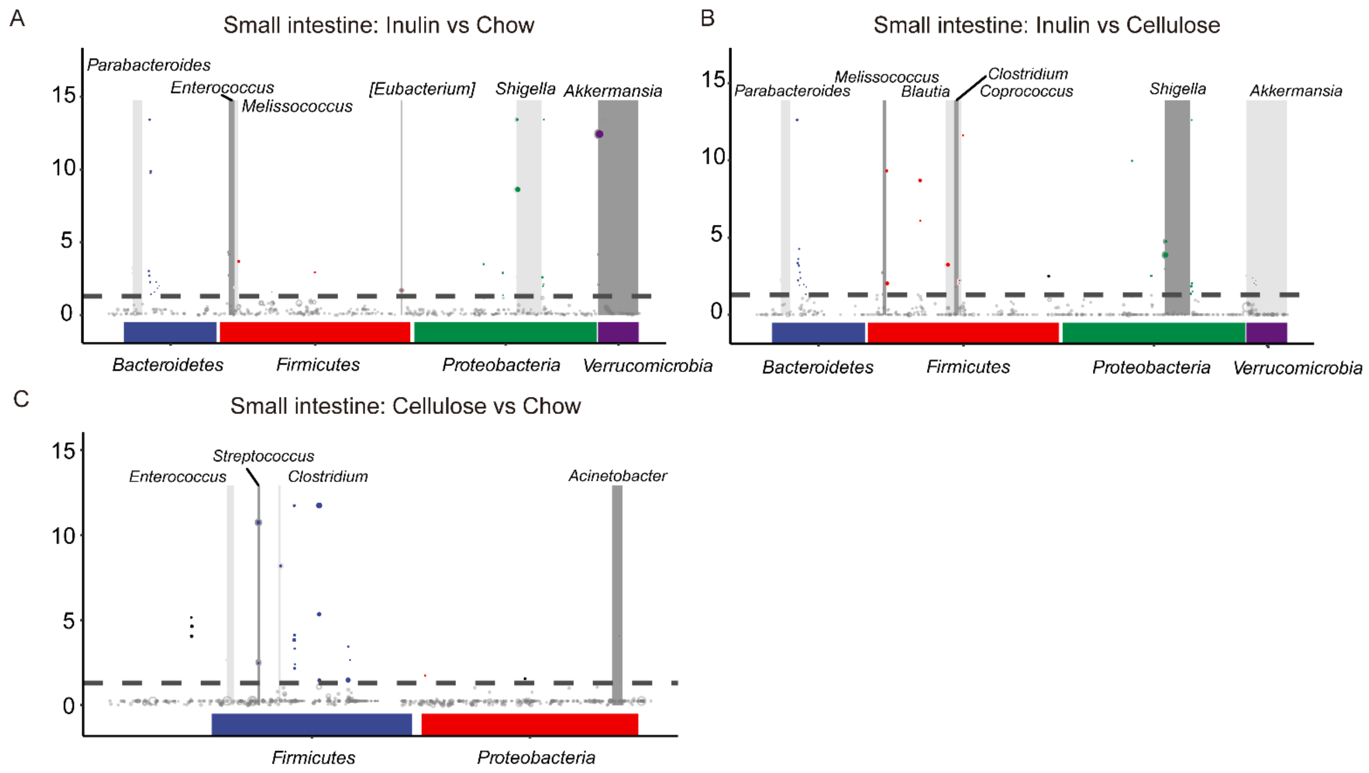

Figure S10. MetagenomeSeq analyses of the microbiota from the small intestine of AAA mice fed with different diets. **(a)** The comparison of gut microbiome from small intestine between inulin and chow diet (n = 6). **(b)** The comparison of gut microbiome from the small intestine between inulin and cellulose diet (n = 6). **(c)** The comparison of gut microbiome from the small intestine between cellulose and chow diet (n = 6).

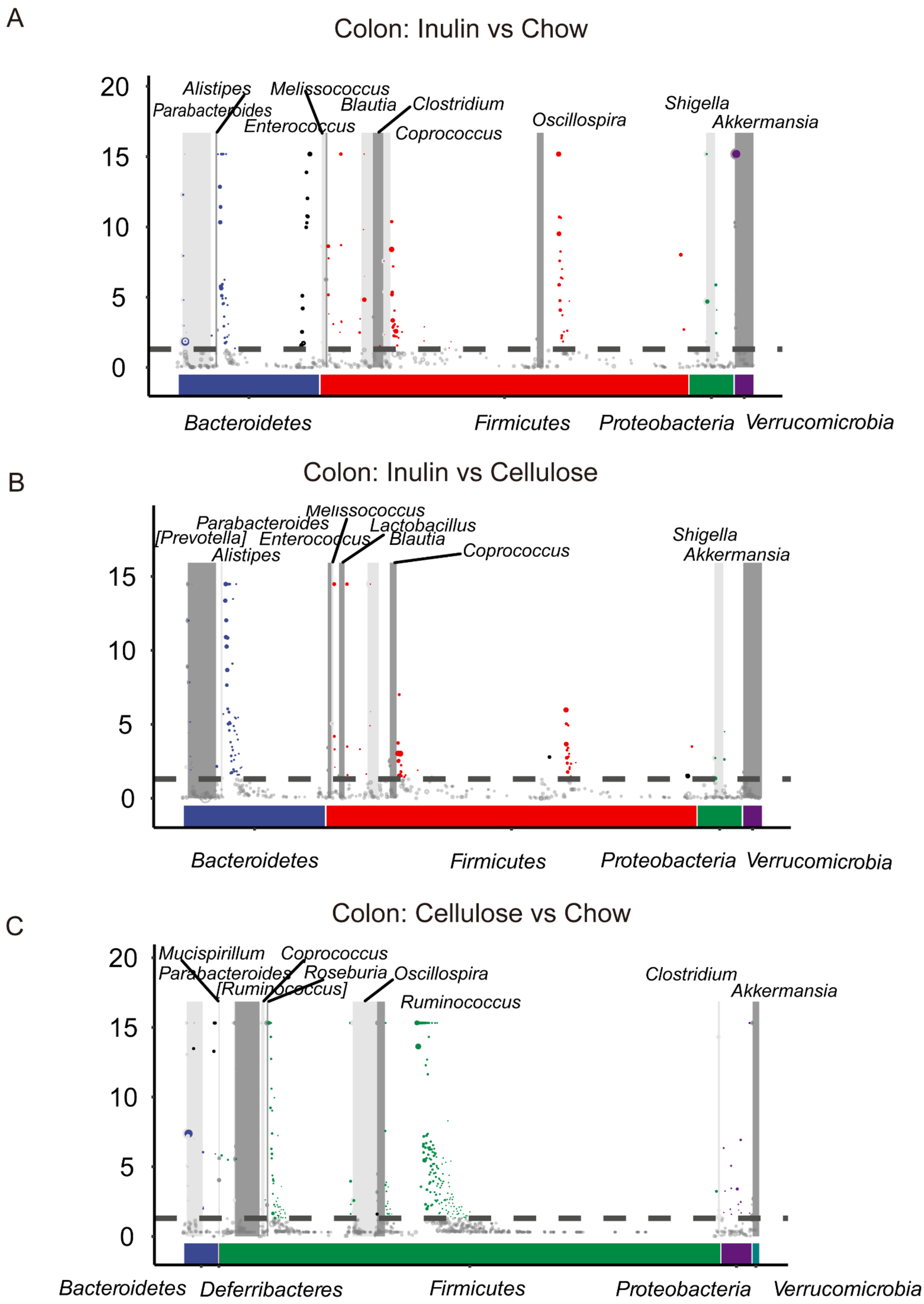

Figure S11. MetagenomeSeq analyses of the microbiota from the colon of AAA mice fed with different diets. **(a)** The comparison of gut microbiome from colon between inulin and chow diet (*n* =

6). **(b)** The comparison of gut microbiome from the colon between inulin and cellulose diet (*n* = 6). **(c)** The comparison of gut microbiome from the colon between cellulose and chow diet (*n* = 6).

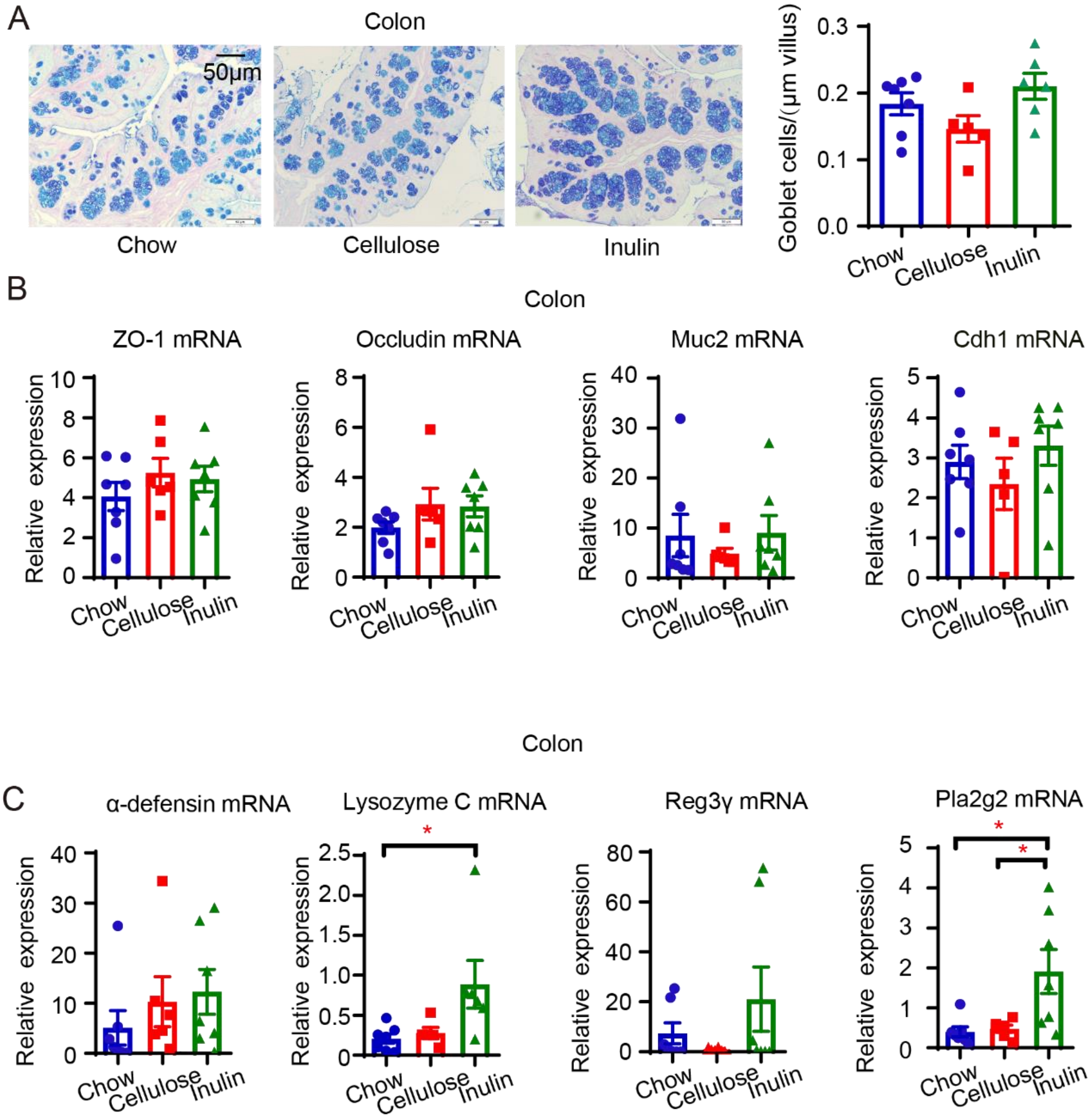

Figure S12. Effects of the inulin diet on the colon barrier of AAA mice. **(a)** Representative Periodic Acid Schiff-Alcian Blue (PAS-AB) staining images of the colon and the numbers of GCs per μm of villus (*n* = 5–7). Scale bars were depicted as indicated in the images. Relative expression of physical barrier-associated proteins **(b)** (*n* = 5–7) and chemical barrier-associated proteins **(c)** (*n* = 5–7) in the colon of AAA mice. * $P < 0.05$. One-way ANOVA followed by Tukey's multiple comparisons test or Kruskal-Wallis followed by Dunnett's multiple comparisons test.

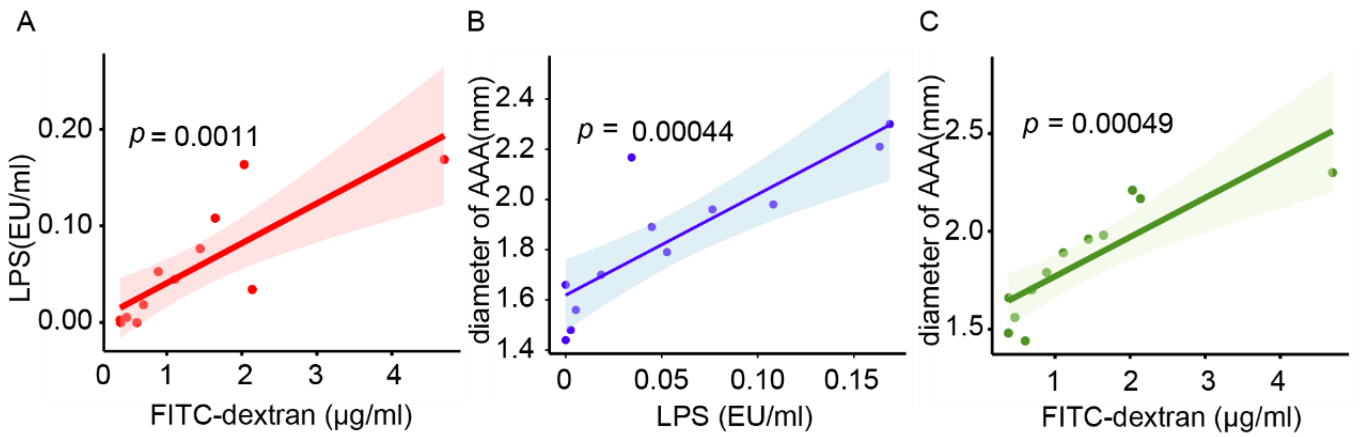

Figure S13. Pearson correlation analyses between intestinal permeability, plasma LPS, and aneurysm diameter. Pearson correlation analyses between intestinal permeability and plasma LPS levels **(a)** (n = 12, R^2 = 0.6746, P = 0.0011), plasma LPS levels and aneurysm diameter **(b)** (n = 12, R^2 = 0.7247, P = 0.00044), intestinal permeability and aneurysm diameter **(c)** (n = 12, R^2 = 0.7196, P = 0.00049).

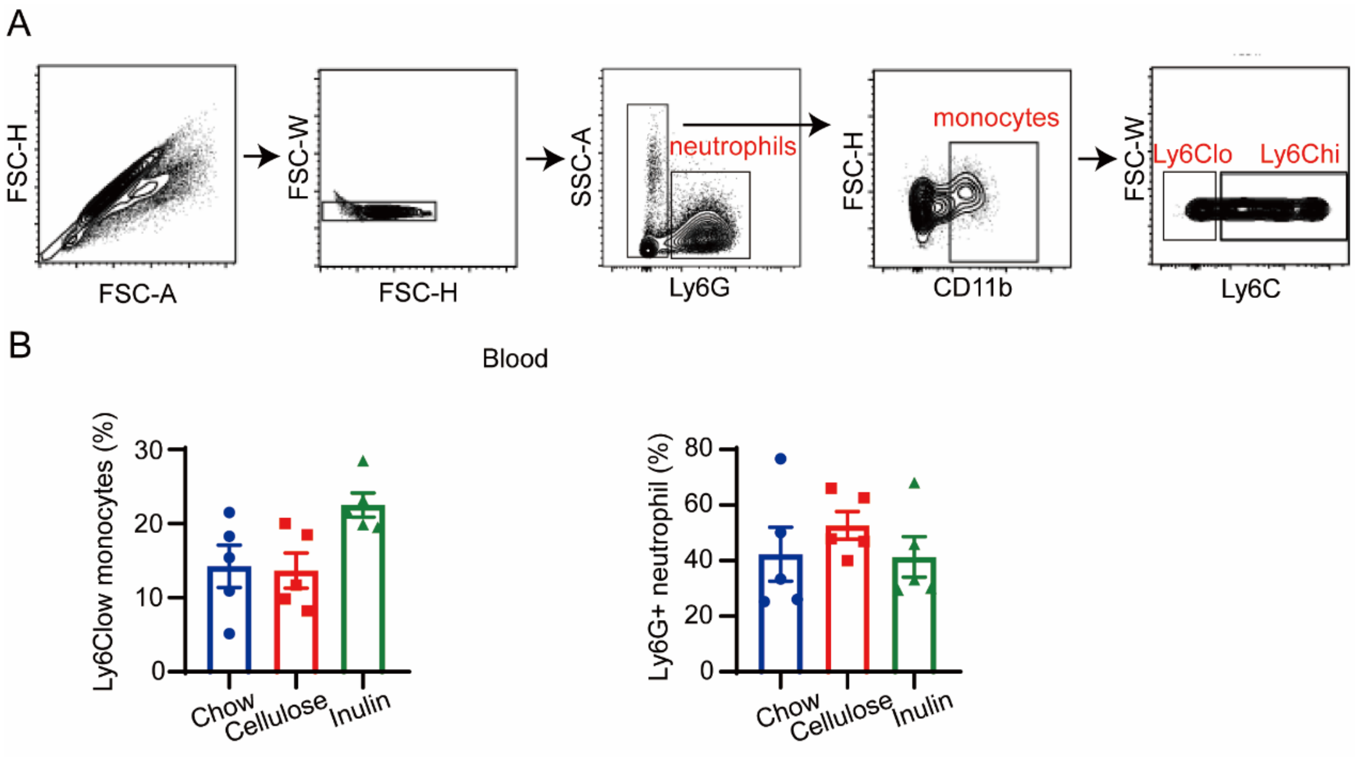

Figure S14. Inulin diet has no impact on Ly6C^{low} monocytes and neutrophils in AAA mice. **(a)** Flow cytometry strategy for the detection of neutrophils, Ly6C^{low} monocytes and Ly6C^{hi} monocytes. **(b)** The percentages of Ly6C^{low} monocytes and neutrophils in the blood of AAA mice (n = 5). One-way ANOVA followed by Tukey's multiple comparisons test.

Table S1. The ingredients of diet for mice.

Product#	D10012M		RD18013004		RD18013005	
	5% Cellulose		15% Cellulose		15% Inulin	
	gm%	kcal%	gm%	kcal%	gm%	kcal%
Protein	14	15	13	15	13	15
Carbohydrate	73	76	65	76	65	76
Fat	4	9	4	9	4	9
Total		100		100		100
kcal/gm	3.8		3.4		3.4	
Ingredient	gm	kcal	gm	kcal	gm	kcal
Casein	140	560	140	560	140	560
L-Cystine	1.8	7.2	1.8	7.2	1.8	7.2
Corn Starch	495.692	1982.768	495.692	1982.768	495.692	1982.768
Maltodextrin 10	125	500	125	500	125	500
Sucrose	100	400	100	400	100	400
Inulin	0	0	0	0	170	0
Cellulose, BW200	50	0	170	0	0	0
Soybean Oil	40	360	40	360	40	360
t-Butylhydroquinone	0.008	0	0.008	0	0.008	0
Mineral Mix S10022M	35	0	35	0	35	0
Vitamin Mix V10037	10	40	10	40	10	40
Choline Bitartrate	2.5	0	2.5	0	2.5	0
FD&C Yellow Dye	0	0	0.025	0	0	0
FD&C Red Dye	0	0	0.025	0	0.025	0
FD&C Blue Dye	0	0	0	0	0.025	0
Total	1000	3850	1352.05	3850	1352.05	3850
Cellulose, %	5		15			
Inulin, %					15	

Key Resources Tables

Antibody information.

Immunostaining antibodies	Dilutions	Concentrations	Catalog numbers	Company names and addresses
CD3	1:100	2 μg/ml	ab5690	Abcam, Cambridge, UK
CD68	1:100	4.7 μg/ml	ab283654	Abcam, Cambridge, UK
MMP2	1:100	10 μg/ml	ab37150	Abcam, Cambridge, UK
MMP9	1:100	10 μg/ml	ab38898	Abcam, Cambridge, UK
Rabbit IgG, polyclonal isotype control	-	Same concentration as the corresponding antigen specific IgG	ab37415	Abcam, Cambridge, UK
Goat Anti-Rabbit IgG H&L(HRP)	1:2000	1 μg/ml	ab205718	Abcam, Cambridge, UK
FACS antibodies	Dilutions	Concentrations	Catalog numbers	Company names and addresses
PerCP/Cy5.5-anti-CD11b	1:80	2.5 μg/ml	101228	BioLegend, CA, USA
FITC-anti-CD45	1:200	5 μg/ml	103108	BioLegend, CA, USA
PEcy7-anti-Ly6C	1:400	0.5 μg/ml	128018	BioLegend, CA, USA
APC/cy7-anti-Ly6G	1:80	2.5 μg/ml	127624	BioLegend, CA, USA
APC-anti-I-A/I-E	1:80	2.5 μg/ml	107614	BioLegend, CA, USA
PEcy7-anti-CD206	1:80	2.5 μg/ml	141720	BioLegend, CA, USA
BV421-anti-F4/80	1:80	2.5 μg/ml	123131	BioLegend, CA, USA
BV605-anti-CCR2	1:40	5 μg/ml	150615	BioLegend, CA, USA
PE/Cy7 Rat IgG2a κ isotype antibody	1:80	2.5 μg/ml	400522	BioLegend, CA, USA
APC Rat IgG2b κ isotype antibody	1:80	2.5 μg/ml	400612	BioLegend, CA, USA

Reagent information.

Enzymes	Concentrations	Catalog numbers	Company names and addresses
Collagenase from Clostridium histolytic Type XI	190 U/ml	C7657	Sigma–Aldrich, Saint Louis, MO
Hyaluronidase from bovine testes	120 U/ml	H3506	Sigma–Aldrich, Saint Louis, MO
Collagenase type I	2 mg/ml	C0130	Sigma–Aldrich, Saint Louis, MO
Deoxyribonuclease I from bovine pancreas	0.5 mg/ml	D4527	Sigma–Aldrich, Saint Louis, MO
Elastase from porcine pancreas	10 mg/mL	E1250	Sigma–Aldrich, Saint Louis, MO
Chemicals	Concentrations	Catalog numbers	Company names and addresses
Buprenorphine	0.1 mg/kg	B-044	Sigma–Aldrich, Saint Louis, MO
Isoflurane	2%, 4%	R510-22	RWD, Shen Zhen, China
FITC-dextran	500 mg/kg	60842-46-8	Sigma–Aldrich, MO, USA
M-CSF	50ng/ml	SRP3221	Sigma–Aldrich, MO, USA
	10 ng/ml	402-ML-100/CF	R&D Systems, Minneapolis, MN
Commercial Kits		Catalog numbers	Company names and addresses
Alcian Blue Periodic Acid Schiff (AB-PAS) Stain Kit	-	G1285	Solarbio, Beijing, China
Cholesterol Quantitation Kit	-	MAK043	Sigma–Aldrich, Saint Louis, MO
Triglyceride Quantitation Kit	-	MAK266	Sigma–Aldrich, Saint Louis, MO
HDL and LDL/VLDL Quantitation Kit	-	MAK045	Sigma–Aldrich, Saint Louis, MO
IL-1β enzyme-linked immunosorbent assay kits	-	BMS6002	Invitrogen, Carlsbad, CA
Kinetic Chromogenic LAL Assay	-	50-650U	Lonza, Basel, Switzerland
DNeasy Blood & Tissue Kit	-	69504	Qiagen, Germany
Others		Catalog numbers	Company names and addresses
LSM-Lymphocyte Separation Medium	-	0850494X	MP Biomedicals, CA
RBC lysis buffer	-	420302	BioLegend, San Diego, CA
Precision Count Beads™	-	424902	BioLegend, San Diego, CA
TRIzol Isolation Reagent	-	15596018	Invitrogen, Carlsbad, CA
PrimeScript™ RT Master Mix	-	RR036A	Takara, Japan
SYBR Green Master Mix	-	RR066A	Takara, Japan
Elastica van Gieson (EVG) staining kit	-	115974	Sigma–Aldrich, Saint Louis, MO
Bacteroides fragilis	-	336948	BNCC, Beijing, China
Akkermansia muciniphila	-	24917	CICC, Shanghai, China

RT-PCR primers.

GAPDH	F:5′-AAATGGTGAAGGTCGGTGTGAAC
	R:5′-CAACAATCTCCACTTTGCCACTG
ZO-1	F:5′-AATCGATGCTGACCTTCTGG
	R:5′-TGAGACGGCTGTCACAACTC
Occludin	F:5′-TGATGTGCATCGCCATATTT
	R:5′-AAGGAAGCGATGAAGCAGAA
Muc2	F:5′-GCTTTGAGACCTGCAGAACC
	R:5′-CGATTTTTGACGTGTTGGTG
Cdh1	F:5′-AGACTTTGGTGTGGGTCAGG
	R:5′-CATGCTCAGCGTCTTCTCTG
α-defensin	F:5′-GGCTGTGTCTGTCTCTTTTGG
	R:5′-CAGCATCAGTGGCCTCAGTA
lysozyme	F:5′-GAGACCGAAGCACCGACTATG
	R:5′-CGGTTTTGACATTGTGTTCGC
Reg3γIII	F:5′-ATGCTTCCCCGTATAACCATCA
	R:5′-GGCCATATCTGCATCATACCAG
Pla2g2	F:5′-TGCTGGCCGGTATAACTGC
	R:5′-CTGTGGCATCTTTGGGTTGC
